# Single cell characterization of blood and expanded regulatory T cells in autoimmune polyendocrine syndrome type 1

**DOI:** 10.1016/j.isci.2024.109610

**Published:** 2024-03-27

**Authors:** Thea Sjøgren, Shahinul Islam, Igor Filippov, Adrianna Jebrzycka, André Sulen, Lars E. Breivik, Alexander Hellesen, Anders P. Jørgensen, Kari Lima, Liina Tserel, Kai Kisand, Pärt Peterson, Annamari Ranki, Eystein S. Husebye, Bergithe E. Oftedal, Anette S.B. Wolff

**Affiliations:** 1 Department of Clinical Science, University of Bergen, Bergen, Norway; 2 Department of Medicine, Haukeland University Hospital, Bergen, Norway; 3 QIAGEN Aarhus A/S, Aarhus, Denmark; 4 Department of Endocrinology, Oslo University Hospital, Oslo, Norway; 5 Department of Medicine, Akershus University Hospital, Lørenskog, Norway; 6 Institute of Biomedicine and Translational Medicine, University of Tartu, Tartu, Estonia; 7 Department of Dermatology, Allergology and Venereology, University of Helsinki and Helsinki University Hospital, Inflammation Centre, Helsinki, Finland

**Keywords:** Health sciences, Immunology, Components of the immune system, Proteomics, Transcriptomics

## Abstract

Immune tolerance fails in autoimmune polyendocrine syndrome type 1 (APS-1) because of *AIRE* mutations. We have used single cell transcriptomics to characterize regulatory T cells (Tregs) sorted directly from blood and from *in vitro* expanded Tregs in APS-1 patients compared to healthy controls. We revealed only CD52 and LTB (down) and TXNIP (up) as consistently differentially expressed genes in the datasets. There were furthermore no large differences of the TCR-repertoire of expanded Tregs between the cohorts, but unique patients showed a more restricted use of specific clonotypes. We also found that *in vitro* expanded Tregs from APS-1 patients had similar suppressive capacity as controls in co-culture assays, despite expanding faster and having more exhausted cells. Our results suggest that APS-1 patients do not have intrinsic defects in their Treg functionality, and that their Tregs can be expanded *ex vivo* for potential therapeutic applications.

## Highlights

• Tregs from APS-1 patients show a higher proliferative capability than healthy controls
• Expanded Tregs from APS-1 patients are biologically active, but are more exhausted
• The genes *CD52*, *LTB* and *TXNIP* have a deviant expression in APS-1 patients’ Tregs
• Some APS-1 patients have a more restricted TCR repertoire than healthy controls

## Graphical abstract

**Figure:**
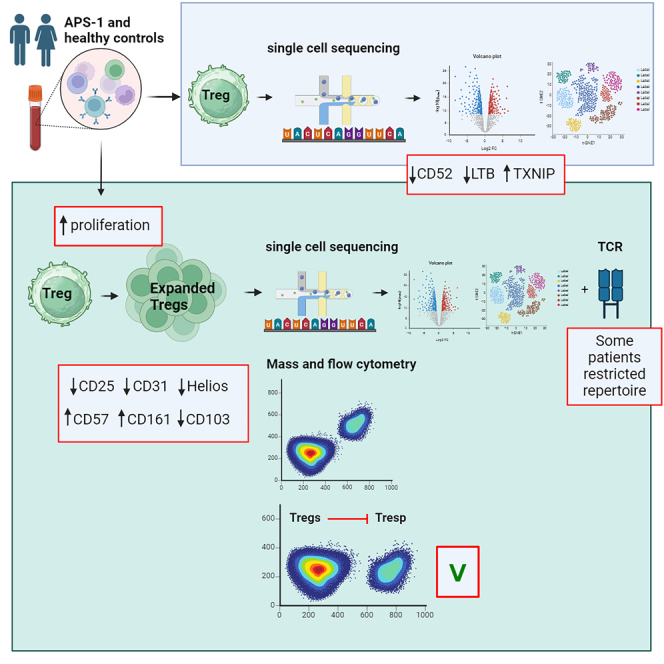

## Introduction

Autoimmune polyendocrine syndrome type 1 (APS-1) is characterized by multiple endocrine and ectodermal manifestations, in addition to chronic *Candida albicans* infections. This rare disease is caused by mutations in the *Autoimmune Regulator* (*AIRE*) gene,^1^^,^^2^^,^^3^ encoding a protein mainly expressed in medullary thymic epithelial cells (mTECs).^4^ Here, it mediates the expression of otherwise tissue-restricted antigens, presenting them to developing T cells and promoting negative selection.^5^^,^^6^^,^^7^ Expression of AIRE has also been noted in a distinct population of peripheral dendritic cells.^8^^,^^9^ AIRE has furthermore been implicated in the generation and recirculation of regulatory T cells (Tregs),^10^^,^^11^^,^^12^ suggesting a checkpoint role for this transcription factor also in peripheral tolerance. Tregs are characterized by stable expression of FOXP3, and are mainly generated in the thymus, but can be induced in the periphery, depending on the cytokine milieu.^13^^,^^14^^,^^15^^,^^16^^,^^17^^,^^18^ The mechanisms of action include both antigen-dependent and bystander-effects, exemplified by depriving effector T cells of IL2, inhibition of the co-stimulatory signal needed for complete T cell activation, and production of inhibitory cytokines, such as IL10, IL35 and TGF-β.^19^^,^^20^^,^^21^

A variety of autoimmune disorders are found with impaired Tregs, ranging from monogenic Tregopathies caused by mutations in Treg regulators like *FOXP3*, *CTLA4*, *WASP*, *CD25*, *STAT5*, and *BACH2*,^22^^,^^23^^,^^24^^,^^25^^,^^26^^,^^27^^,^^28^^,^^29^^,^^30^^,^^31^ to common diseases like type 1 diabetes, multiple sclerosis, rheumatoid arthritis and polyendocrine syndromes.^32^^,^^33^^,^^34^^,^^35^ Studies in mice have revealed the role of the Aire protein in influencing Treg development and function, and suggested possible interactions for humans. For example, mice that are double-deficient of Aire and Foxp3 develop severe autoimmunity early in life,^36^ and are more severely affected than both Aire and Foxp3 single-knockout mice. Aire also affects the generation of a specific subtype of Tregs in the perinatal period which may be important for preventing autoimmunity.^37^^,^^38^^,^^39^ Malchow et al. further showed that the most frequent Treg specificities develop independently of Aire, while a few are Aire dependent and these may be involved in driving autoimmunity in Aire knockout mice.^39^ Similarly, in humans, patients with APS-1 have fewer Treg clones of common TCR-β sequences, which instead were found in the conventional T cell compartment.^40^ Several studies have found reduced numbers of circulating Tregs with impaired suppressive or tissue homing capacities in APS-1 patients,^41^^,^^42^^,^^43^^,^^44^^,^^45^ but it is still debated whether APS-1 is a functional Tregopathy. Nonetheless, the Treg deficiency in these patients suggests that enhancing or modifying Tregs could be a potential therapeutic strategy to restore immunological tolerance.

Despite increased knowledge on Tregs and their crucial role in avoiding autoimmune disease, we are still lacking a detailed understanding of human Treg mechanisms and function, especially at the single-cell level. Based on AIRE’s possible involvement in the induction of thymic CD4+CD25+FOXP3+ Tregs, we have here undertaken a detailed analysis of freshly sorted human blood Tregs in APS-1. As Tregs are scarce in number, additional analyses were conducted on expanded polyclonal Tregs including a rigorous suppression assay, proteomic characterization by mass and flow cytometry, single-cell analysis of the transcriptome and TCR repertoire and Treg-specific cytokines. This is, to our knowledge, the first time that single-cell sequencing has been performed to study Tregs from APS-1 patients.

## Results

### Single cell transcriptomics of freshly sorted Tregs from APS-1 patients

**Figure 1:**
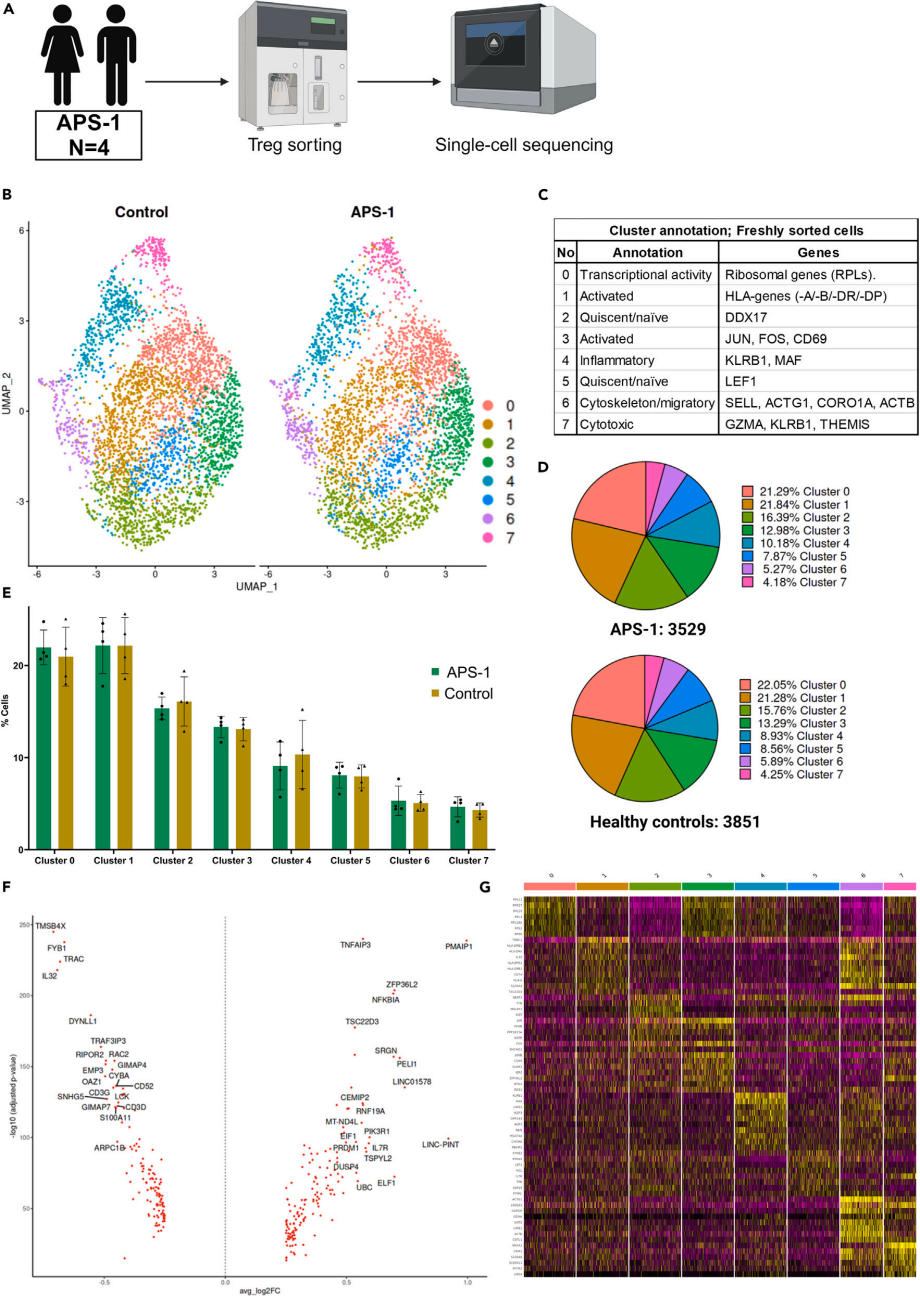
Single-cell transcriptomic profiles of freshly sorted Tregs from four APS-1 patients and four healthy controls (A) Method and number of patients. (B) UMAP plot of global single-cell sequencing data for patients and controls, identifying eight distinct clusters of Tregs. (C) Cluster annotations with some of the most expressed genes in each cluster. (D) Pie charts of cluster frequencies in APS-1 patients and healthy controls. (E) Bar plots of cluster frequencies per individual. Statistical testing was performed by the Mann-Whitney non-parametric t-test. Standard deviation is included for each bar. Significance level: p < 0.05∗. (F) Volcano plot of the most differentially expressed genes (DEGs) (−0.2 > log2FC > 0.2) between APS-1 and healthy controls, where log2FC > 0 represents upregulated genes and log2FC < 0 represents downregulated genes in APS-1. (G) Heatmap of the most differentially expressed genes (DEGs) (−0.2 > log2FC > 0.2) between APS-1 and healthy controls; reported as the average, normalized SCT expression value of the genes, taking into consideration all the cells that express a specific gene. The figure was made using BioRender (Biorender.com). *See also*Figures S1 and S7*and*Table S5.

To investigate Tregs at the single cell level, CD4+CD25+CD127^low^ cells were sorted from four Finnish APS-1 patients and matched healthy controls, libraries were made and global single cell expression was analyzed (Figure 1A). Forced clustering resulted in eight clusters characterizing the cell cohort (Figures 1B and 1C). The clusters were equally distributed between APS-1 patients and controls (Figures 1D and 1E). Therefore, we chose to compare the gene expression of all Tregs between APS-1 patients and healthy controls. We identified 277 genes that were significantly different, although with moderate log2FC changes, i.e., between (1.00) and (−0.71) (Figures 1F and 1G, and Table S5). Pathway enrichment analysis of differentially expressed genes pointed to cell movement and T cell signaling as mildly disturbed events in APS-1s′ Tregs (Figure S1A). We further found that some pathways were inhibited (α-synuclein (SNCA), the TEAD family of transcription factors 1 (TEAD1) and the ubiquitin protein ligase E3 component n-recognin 5 (UBR5) pathways), and some were activated (signal transducer and activator of transcription (STAT) members 3 and 6) in patients by target network analysis (Figure S1B).

### *In vitro* Treg expansion of cells from APS-1 patients and healthy controls

**Figure 2:**
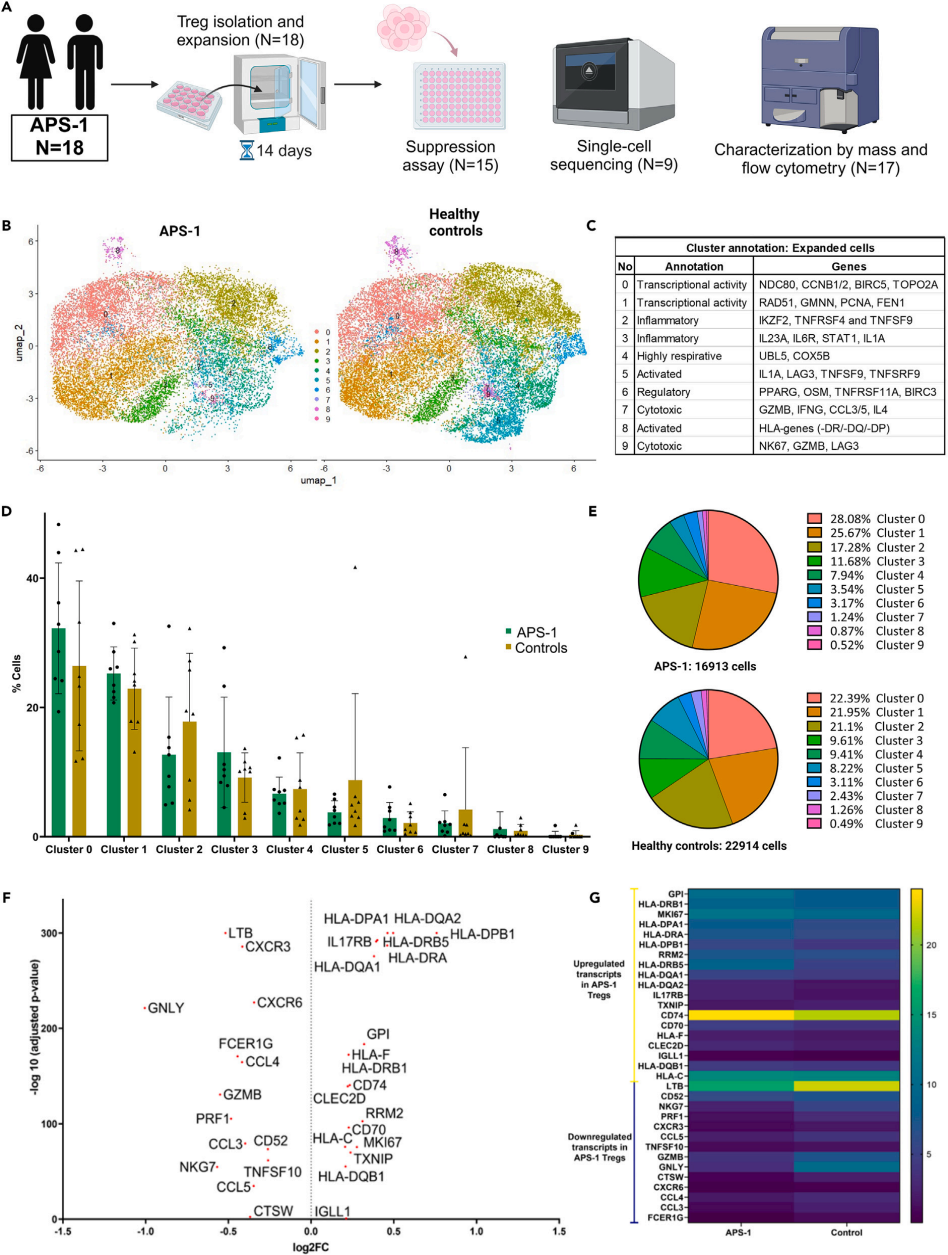
Single-cell transcriptomic profiles of *in vitro* expanded Tregs from eight APS-1 patients and eight healthy controls (A) Method and number of patients. (B) UMAP plot of immune panel single-cell sequencing data for patients and controls, identifying ten distinct clusters of Tregs. (C) Cluster annotations with some of the most expressed genes in each cluster. (D) Bar plots of cluster frequencies per individual. Statistical testing was performed by the Mann-Whitney non-parametric t-test. Standard deviation is included for each bar. Significance level: p < 0.05∗. (E) Pie charts of cluster frequencies in APS-1 patients and healthy controls. (F) Volcano plot of the most differentially expressed genes (DEGs) (−0.2 > log2FC > 0.2) between APS-1 and healthy controls, where log2FC > 0 represents upregulated genes and log2FC < 0 represents downregulated genes in APS-1. (G) Heatmap of the most differentially expressed genes (DEGs) (−0.2 > log2FC > 0.2) between APS-1 and healthy controls; reported as the average, normalized SCT expression value of the genes, taking into consideration all the cells that express a specific gene. The figure was made using BioRender (Biorender.com). *See also*Figures S2–S7*and*Tables S2, S3, S6, S7, and S8.

APS-1 is a rare disorder (prevalence 1:100000 in the Nordic countries,^46^ except for Finland, with a prevalence of 1:25000) and patients are dispersed all over the country. As Tregs are a scarce cell population in blood, we were unable to obtain enough Tregs from an adequate number of persons to conduct further immunophenotypic and functional assays on freshly sorted cells. Therefore, we chose to explore the potential for Tregs from APS-1 patients to expand in response to polyclonal activation in culture, and to characterize such expanded Tregs at the single cell level using both transcriptomic and proteomic tools (Figure 2A).

After isolation (day 0), the CD4+CD25+CD127^low^ expanded Treg fraction contained a mean of 250000 cells (range 150000–450000 cells) for patients and 300000 cells (range 150000–720000 cells) for controls. To obtain enough cells for all downstream analysis, the up-concentrated Tregs were expanded for 14 days with a mean fold increase of 52 (range 15–132) for patients and 37 (range 7–68) for controls. We observed that more than 90% of the live cells expressed FOXP3 (range patients 94.4–99.2%, range controls 93.1–98.2%, p = 0.0963) and that more than 95% of the CD4+ cells expressed FOXP3 (range patients 97.5–99.7%, range controls 98.3–99.6%, p = 0.676, Figure S2).

T cells generally have high plasticity and can differentiate into various subsets depending on the activation signals they receive. However, unregulated activation may lead to unwanted differentiation outcomes. Cells from APS-1 patients may also have intrinsic factors that bias them toward certain T cell subsets. Therefore, we analyzed the expression of the linage defining genes *FOXP3* (master regulator of Tregs), *RORγt* (master regulator of Th17-cells), *GATA3* (master regulator of Th2 cells), *Tbet* (master regulator of Th1 cells), and the functional markers *CCR4* and *CXCR3* from all subjects. All of these markers were expressed in expanded Tregs from all subjects, but there were no differences between patients and healthy controls (Figure S3A). Among the markers, *CCR4* and *CXCR3* had the highest expression, followed by *GATA3* and *FOXP3* (Figure S3B).

### Single-cell transcriptomic characteristics of expanded Tregs

We performed single-cell transcriptome sequencing using a targeted Human Immunology Panel to compare the expanded Treg population from eight APS-1 patients and eight healthy controls (Figure 2A). The cells in both cohorts were distributed across all cell cycle phases (G1, G2M and S) with no significant difference (Tables S3 and S4A). We examined the expression of Treg defining molecules (*FOXP3, IL2RA, IL2RB, TNFRSF1B, TNFRSF4, SPOCK2, CD28, CD3D, CD3G, and CD4*) in individual samples and found that most cells expressed them adequately (Figures S4B and S4C). However, some carry-over natural killer (NK) cells (positive for *FCER1GA/3A, KIR2DL3/2DL1, STSW, GNLY, CCL3/4, TRDC* and *PRF1*) and B cells (positive for *Jchain, CCL17/22, IGHM/KC/HG1/HA1, CD79A, MS4A1* and *CXCL10*) could be seen (Table S3). Patients and controls generally contained >91% Tregs based on this analysis, with two strange healthy control outliers, containing high proportions of NK-cells (52%) and Th17-cells (26%) (based on high expression of IL22 and IL17F), respectively, and one patient had a high number of B cells (13%) (Table S3). We performed differential expression analysis with and without the outliers, but we only report the results with all samples included, as the main differentially expressed genes were consistent in both cases.

The APS-1 and healthy controls did not cluster differently in a uniform manifold approximation and projection (UMAP) for dimension reduction. The single-cell profiles of the two groups were overlaid and forced cluster analysis identified ten distinct clusters (Figures 2B and S4). Twenty of the most expressed genes in each group is provided in Table S6, and the clusters were annotated accordingly, although only with a few of the clusters revealing clear mechanistic relevance (Figure 2C). The distribution of the expanded Treg clusters were overall similar for patients and controls, but patients had some fewer cells in clusters 2, 5 and 7, while controls had fewer cells in clusters 0, 1, and 3 (Figures 2D and 2E), without reaching statistically significance. Of note is that the downregulated cluster 2 contained the genes *IKZF2*, *TNFRSF4* and *TNFSF9*, all being part of the TNF superfamily, and downregulated cluster 5 contained several markers associated with T cell activation and regulation, including *SOD1, IL1A, LAG3, TNFSF9, TNFRSF9* and *CORO1A*, in addition to members of the NFKB signaling pathway (*CXCL8, NFKBIA, BCL2A1*) being involved in interferon responses. Cluster 7, which was also decreased in APS-1 patients, contained genes encoding T cell-attracting chemokines (CCLs), cytokine activation pathways including IL4, IL5, CCL3, NAMPT, CCL5 and LIF and genes in the JAK-STAT pathway. Additionally, cluster 7 included the gene *GZMB*, indicating granzyme-mediated cytotoxicity (Table S7). A specific outlier is cluster 8, containing cells from only one patient and one control.

Focusing on the global transcriptome and not clusters, a few genes were found to be significantly differentially expressed between patient and healthy control expanded Tregs (Table S8), although the magnitudes of the differences were moderate, ranging from log2FC (−1.01) to (0.76) (p < 0.001). Among the top differentially expressed genes (−0.2>log2FC > 0.2), several *HLA*-genes and the proliferation marker *MKI67* were significantly upregulated in APS-1 patients (Figures 2F and 2G). Genes that were downregulated in APS-1 patients included *chemokine ligands* (*CCL*) *3*, *CCL4* and *CCL5*, *chemokine receptors CXCR3* and *CXCR6*, in addition to *granzyme* (*GZM*) *B* and *TNFSF10* (Figures 2F and 2G). A heatmap of the top 15 differentially expressed genes showed high variability in gene expression among cells and subjects (Figure S5).

Pathway enrichment analysis of differentially expressed genes from the expanded Treg analysis revealed that patients had downregulated pathways related to cytokines, antigen presentation, and T cell subsets, and upregulated pathways related to IL10 and PD1 signaling, which are involved in suppression (Figure S6).

To compare differentially expressed genes from the freshly sorted Tregs and expanded Tregs analysis, we generated a graph in which the log2FC-values from each of the experiments represented one dimension (Figure S7). Applying a filter of |log2FC| > 0.2, only two genes were consistently down-regulated (*CD52* and *LTB*) and one gene up-regulated (*TXNIP*) in APS-1 patients compared to healthy controls. This analysis should be interpreted with caution as it was based on different patient cohorts and protocols.

### The TCR repertoire of expanded Tregs

**Figure 3:**
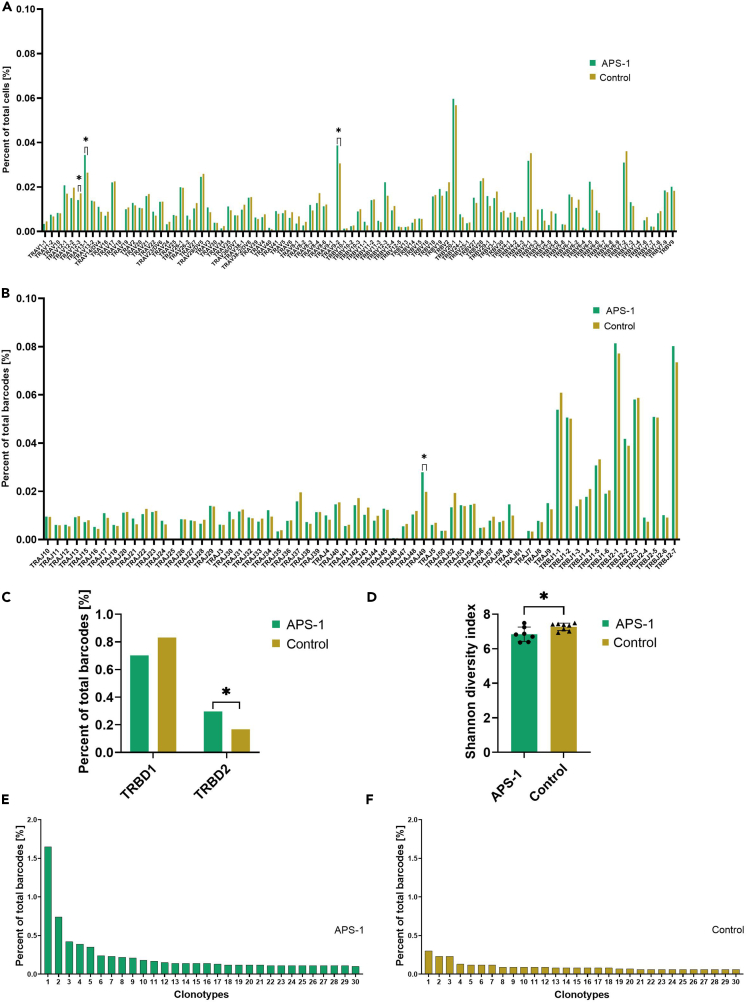
TCR VDJ gene usage analysis of *in vitro* expanded Tregs from seven APS-1 patients and nine healthy controls (A) V gene usage. (B) J gene usage. (C) D gene usage. (D) Summary histogram showing the Shannon diversity index (all clones) of *in vitro* expanded Tregs from seven APS-1 patients and eight healthy controls. ∗Threshold for significance is set to p < 0.05, calculated using a Mann-Whitney test for the Shannon Index. Standard deviations are shown for the bars. Frequencies of top 30 most abundant clonotypes, where clonotype is defined as the combined TRA+TRB amino acid sequences, for (E) APS-1 patients and (F) healthy controls. None of the top 30 clonotypes were shared between the two groups. *See also*Figures S8–S12.

To determine the immunological repertoire of the Tregs, single-cell TCR sequencing was performed on *in vitro* expanded Tregs from nine APS-1 patients and nine healthy controls. After initial quality control and filtering, two samples did not show enough diversity (reads mapped to any V(D)J gene <72%), which was set as one of the prerequisites for analysis, and were thus removed from the dataset. We observed very low overlap between the most frequent clonotypes on the individual level. Moreover, APS-1 patients had some clonotypes dominating to a large extent over others, while healthy controls had a more even distribution of the most expressed clonotypes (Figures 3E and 3F). These dominant clonotypes came from individual patients, which might indicate that distinct APS-1 patients have a restricted TCR repertoire (Figures S8–S12).

To examine the clonotype frequency in the cohorts, we performed an aggregated analysis. For healthy controls, a total number of 46701 cells, 32349 clonotypes and 36688 cells with productive V-J spanning pair were acquired. For APS-1 patients, we obtained a total number of 37679 cells, 24629 clonotypes and 28608 cells with productive V-J spanning pair. VDJ gene usage analysis showed that certain VJ sequences, such as TRAV13-1, TRAV9-2, TRBV12-3 and TRAJ-49 (p < 0.05) were used more frequently in APS-1 patients compared to healthy controls (Figures 3A, 3B, and S8–S10). For D gene usage, healthy controls tended to use TRBD1 more (p = 0.062, Figure 3C), while TRBD2 was significantly more used in APS-1 patients (p = 0.041, Figure 3C).

We compared the clonotype diversity of APS-1 patients and healthy controls using the Shannon Diversity Index and found that patients had a significantly lower mean index, indicating lower diversity (p = 0.0413, Figure 3D). However, there were large variations between individuals in both cohorts, with Shannon diversity indices ranging from 6.4 to 7.5 (mean 6.8; SD 0.4; 1088–4339 total clonotypes >0) in patients and 6.9 to 7.5 (mean 7.3; SD 0.2; 1914–5565 total clonotypes >0) in healthy controls.

### Proteomic characterization of expanded Tregs

**Figure 4:**
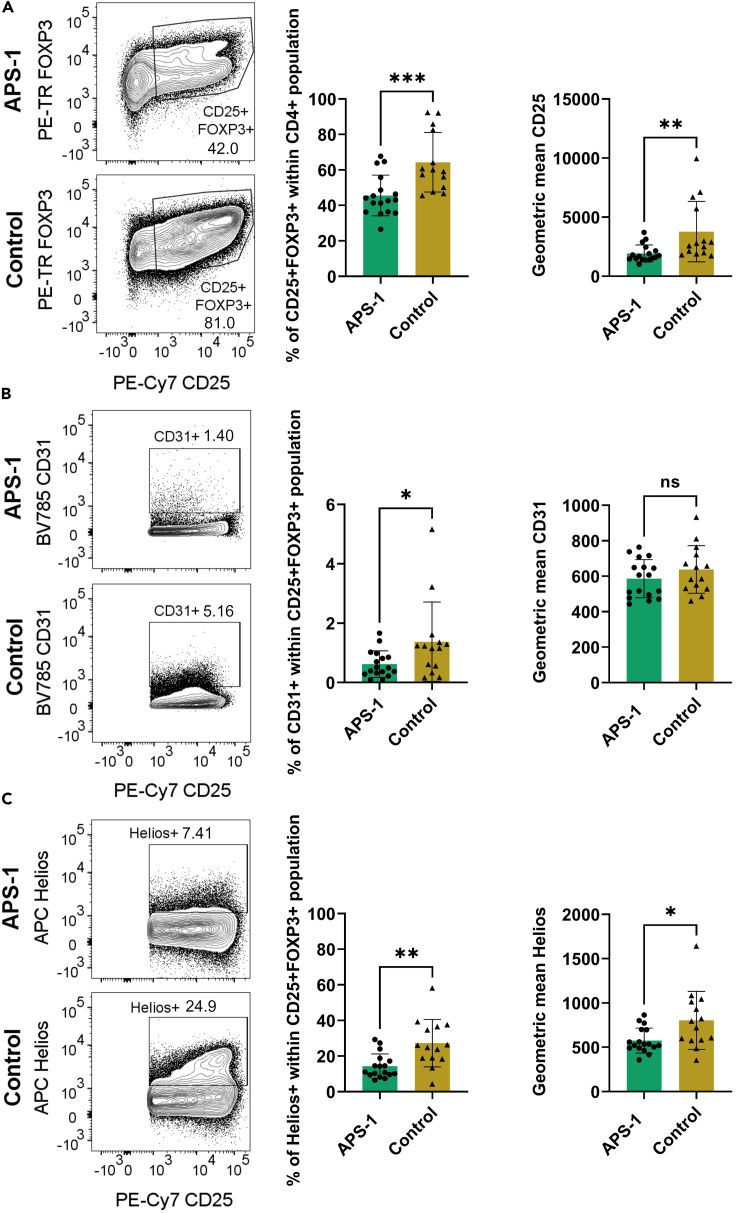
Flow cytometry analysis of FOXP3, CD25, CD31 and Helios expression in CD4+CD25+FOXP3+ cells of expanded Tregs in 17 APS-1 patients and 14 healthy controls (A) Frequency of CD4^+^CD25+FOXP3+ cells, and geometric mean of CD25 within the CD4^+^ population, (B) CD31 within CD4^+^CD25+FOXP3+ cells and (C) Helios within CD4^+^CD25+FOXP3+ cells. ∗p < 0.05, ∗∗p < 0.01 and ∗∗∗p < 0.001. Statistical tests were calculated using an unpaired, parametric t-test. Standard deviations are shown for all bars. *See also*Figures S13 and S14*and*Table S1.

To follow up the transcriptional changes we wanted to determine the levels of important Treg proteins in expanded Tregs using mass and flow cytometry. *In vitro* expanded Tregs from 17 APS-1 patients and 14 healthy controls were first examined for the expression of ten Treg markers using flow cytometry (gating strategy in Figure S13). Although >97% of both patient and control CD4^+^ cells expressed the Treg signature molecule FOXP3, the CD4+CD25+FOXP3+ population, was significantly lower in APS-1 patients compared to healthy donors (mean patients 45.6%, mean controls 64.3%, p = 0.0009, Figure 4A). We further observed a lower geometric mean of the activation marker CD25 within the CD4^+^ population in patients (mean patients 1924, mean controls 3772, p = 0.0078, Figure 4A).

We detected a difference in the expression of the recent-thymic emigrant marker CD31 within CD4+CD25+FOXP3+ cells, where the expression was lower in APS-1 patients compared to healthy controls (mean patients 0.6%, mean controls 1.4%, p = 0.0369, Figure 4B). The geometric mean for this marker was also slightly lower for APS-1 patients in average, but without reaching significance (mean patients 586.5, mean controls 638.0, p = 0.2445, Figure 4B). The intracellular functional marker Helios, encoded by the *IKZF2* gene, was also lower in APS-1 patients’ cells compared to controls (frequencies mean patients 14.3%, mean controls 27.3%, p = 0.0016; geometric mean (mean patients 575.6, mean controls 804.0, p = 0.0143, Figure 4C). There were no differences in expression of the naive Treg marker CD45RA, the activation marker HLA-DR and the Treg functional marker CD39 within the CD4+CD25+FOXP3+ subset between patients and controls (Figure S14).

We used a 27-marker CyTOF panel to complement the flow cytometry analysis in 17 APS-1 patients and 17 healthy controls. The applied antibody panel enabled us to investigate possible contaminating cell subsets within the expanded Treg cohorts as indicated for three samples in the transcriptomic analysis. Seventeen of the chosen markers were important for Tregs, while 10 lineage hallmarks of other cell types were included as “exclusion markers”. After pre-processing, data clean up and batch correction (Figure S15), we gated for CD3+CD4+CD127^low^ cells, as the expression of CD127 is inversely proportional to the expression of FOXP3,^47^ and further as detailed in Figure S16. As expected, monocytes, granulocytes, NK cells and CD8^+^ T cells were overall found to be present in negligible numbers (average frequencies < 2%), based on the expression of the markers CD14, CD66b, CD56 and CD8a (Table S4). However, one control had high CD8 (17.9%) whereas another had a high expression of CD56 (7.2%), both within the CD45^+^ fraction, suggesting some contamination. The average expression of CD19 in patients within the CD45^+^ cell population was 0.5% (range 0–1.7%) and for healthy controls the average was 1.3% (0–6.1%). Even though two control samples exhibited 5% and 6% B cells in the CD45 pool, the overall B cell content was low in each group and there was no pattern of more B cell carry-over in one cohort over the other.

**Figure 5:**
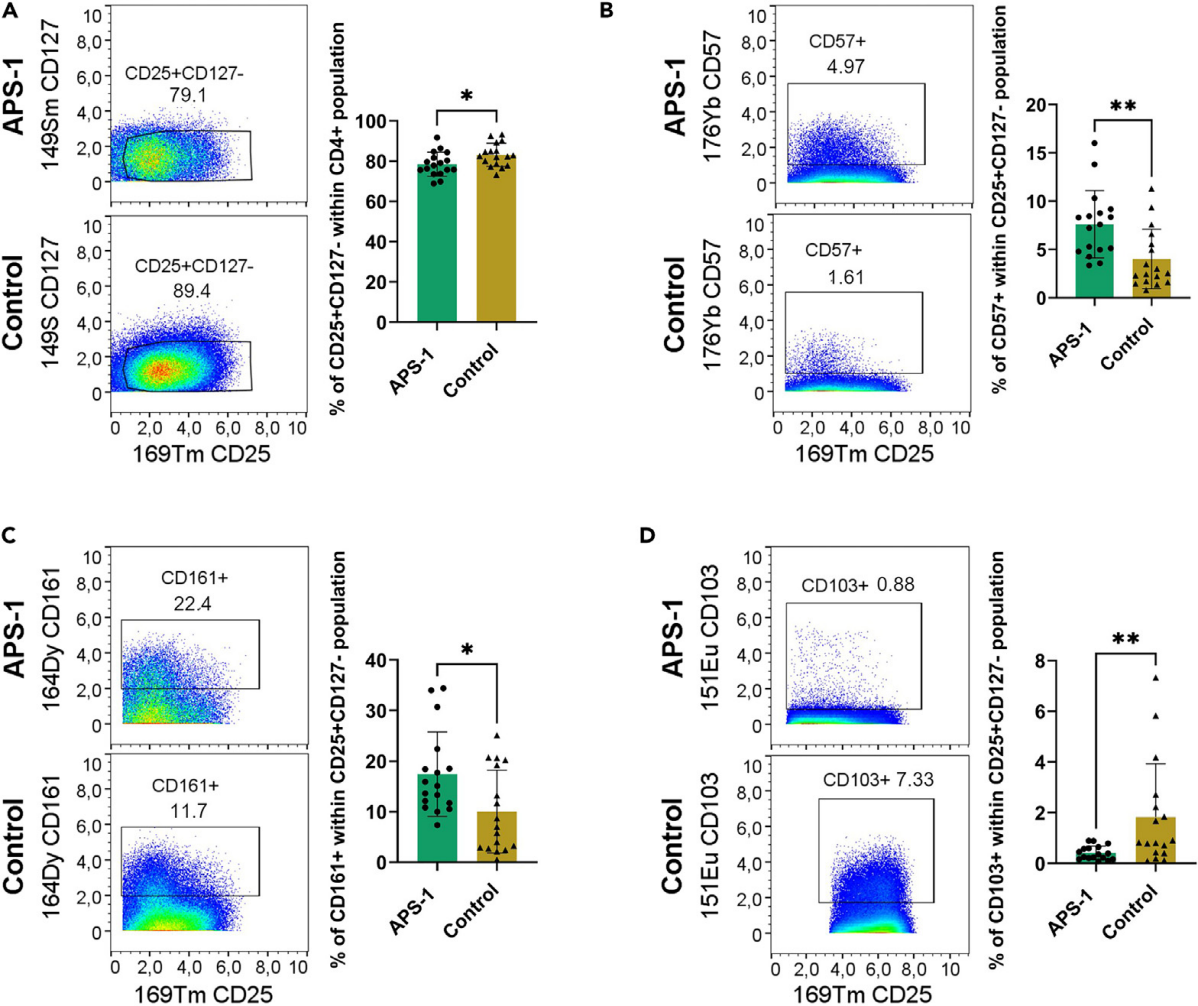
Expression of different T cell markers within the CD4+CD25^+^ CD127^low^ expanded Treg population analyzed by CyTOF in 17 APS-1 patients and 17 healthy controls (A) Frequency of CD4^+^CD25^+^ CD127^low^cells. Expression of (B) CD57, (C) CD161 and (D) CD103 within CD4^+^CD25^+^ CD127^low^ cells. The figures are shown for flow cytometry results of a representative APS-1 patient and healthy control. The p values were calculated using an unpaired, parametric t-test. ∗p < 0.05 and ∗∗p < 0.01. Standard deviations are shown for the bars. *See also*Figures S15–S17*and*Tables S1 and S4.

As for the flow cytometry experiment, the CD4+CD25+CD127^low^ Treg fraction in mass cytometry experiments was significantly lower in APS-1 patients versus controls (mean patients 78.5%, mean controls 83.2%, p = 0.0282, Figure 5A). We also observed a significant upregulation of the markers CD57 (mean patients 7.6%, mean controls 4.0%, p = 0.0032, Figure 5B) and CD161 (mean patients 17.4%, mean controls 10.0%, p = 0.0135, Figure 5C) in the patients, while CD103 (mean patients 0.4%, mean controls 1.8%, p = 0.0096, Figure 5D) was significantly downregulated in CD4+CD25+CD127^low^ cells. We used the expression of the markers CD45RA and CD45RO to differentiate naive and activated Tregs and did not observe differences between patients and controls. CD4+CD25+CD127^low^ cells from both patients and controls showed a low expression of CD45RA (mean patients 2.7%, mean controls 1.2%, p = 0.1793, Figure 5), and as expected, the majority of cells showed a high expression of CD45RO (mean patients 91.3%, mean controls 89.8%, p = 0.4389, Figure S17). For expression of the remaining markers, there were no significant differences between patients and controls (Figure S17).

### Expanded Tregs from APS-1 patients are capable of suppressing T effector cells *in vitro*

As our analysis points to subtle differences between patients and healthy controls, we further set out to assess the polyclonal suppressive capacity of the expanded Tregs in 15 APS-1 patients and 15 healthy controls. To this end, we established an *in vitro* co-culture suppression assay, measuring the ability of Tregs to inhibit CD4+ and CD8+ responder cell (Tresp) proliferation. CellTrace Violet-labeled Tresp cells were co-cultured with Tregs at different ratios for five days and the percentage suppression was calculated for each ratio. Tresp (CD4+CD25−) and expanded Tregs (CD4+CD25+) were distinguished according to CD25 and CellTrace Violet, and the frequency of Tresp cells were used in the calculation of suppressive capability of the expanded Tregs (gating strategy Figure S18).

**Figure 6:**
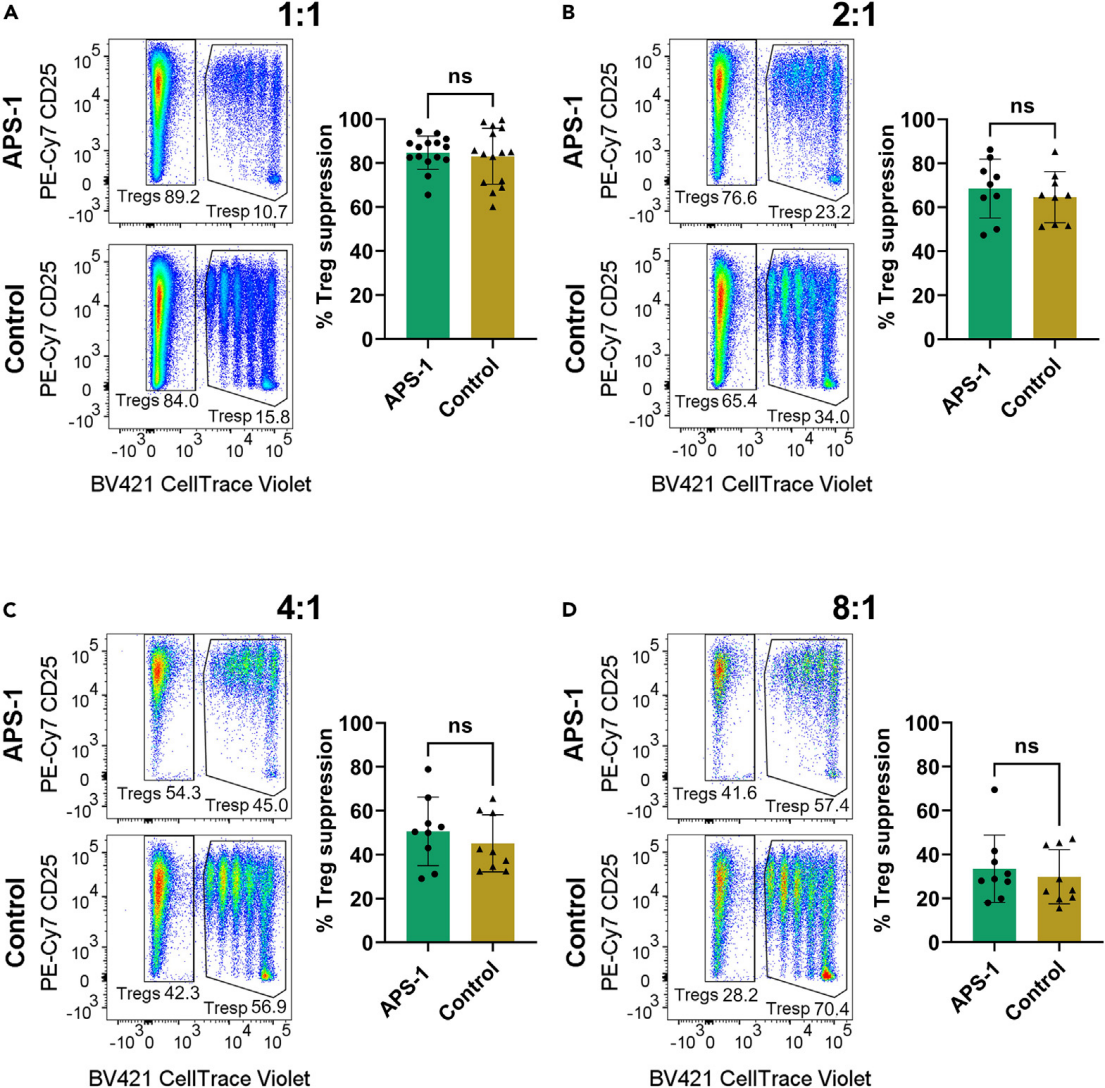
*In vitro* Treg suppression assay for 15 APS-1 patients and 15 healthy controls CellTrace Violet labeled Tresp cells were co-cultured with Tregs at different ratios for 5 days in the presence of anti-CD3/CD28 and IL2. (A) Tresp:Treg 1:1, (B) Tresp:Treg 2:1, (C) Tresp:Treg 4:1 and (D) Tresp:Treg 8:1. The figures show flow cytometry results from a representative APS-1 patient and a representative healthy control for each ratio. P-values were determined by an unpaired, parametric t-test. A p value <0.05 was considered significant. ns; non-significant. Standard deviations are shown for the bars. *See also*Figures S18–S21*and*Table S1.

Four different Tresp:Treg ratios (1:1, 2:1, 4:1 and 8:1) were used initially for patients #5–13 (Table 1) and matched controls, while only 1:1 was evaluated for the remaining patients (Table 1 #14–20). The Treg suppression efficacy ranged from 65.5% to 94.4% (mean 84.7%) for patients and 60.1%–99.6% (mean 83.1%) for controls at 1:1, and then gradually declined for both groups with decreasing amount of expanded Tregs added. For all ratios, the mean suppression was slightly higher for APS-1 patients compared to healthy controls, but the difference was not significantly different (p > 0.05) (Figure 6). We observed similar results for CD8^+^ Tresp suppression (Figure S19). Proliferation and expansion indices for the Tresp:Treg ratios 1:0 and 1:1 within the CD4^+^ responder cell population did not differ between patients and controls (p > 0.05, Figure S20). ELISA assays were performed to measure the concentration of the Treg-specific cytokines IL35, IL10 and TGF-β in suppression assay supernatants. Although IL35 and TGF-β were detectable for all samples, and IL10 for most, no differences in cytokine production between the cohorts were observed (Figure S21).

**Table 1:** Clinical features of the included APS-1 patients

Patient	Sex^a^	Manifestations^b^	*AIRE* mutation
1	F	Autoimmune PAI, CMC, HP, POI, V, GHdef	c.769C>T/c.769C>T
2	F	Autoimmune PAI, CMC, HP, POI	c.769C>T/c.769C>T
3	F	Autoimmune PAI, CMC, HP, POI, HypoT	c.769C>T/c.769C>T
4	F	Autoimmune PAI, CMC, HP, POI, V, Al	c.769C>T/c.769C>T
5	M	T1D, HP, Al, CMC	c.769C>T/c.1249dupC
6	M	CMC	c.769C>T/c.1249dupC
7	F	Autoimmune PAI, CMC	c.879 + 1G>A/c.879 + 1G>A
8	F	HP, B12, POI	c.934G>A dominant
9	M	Autoimmune PAI, HP, CMC	c.22C>T/c.967_979del
10	F	Autoimmune PAI, HP, POI, CMC	c.967-979del13/c.967-979del13
11	M	Autoimmune PAI, CMC	c.967-979del13/c.967-979del13
12	M	Autoimmune PAI, HP, Al, CMC	c.967-979del13/c.967-979del13
13	F	Autoimmune PAI, HypoT, HP, POI, CMC	c.967-979del13/large del
14	M	HP, CMC	c.967-979del13/c.769C>T
15	M	Autoimmune PAI, HypoT, HP, Al, CMC	c.967-979del13/c.769C>T
16	M	Autoimmune PAI, CMC	c.967-979del13/c.967-979del13
17	F	Autoimmune PAI, HyperT, POI	c.1336C>G/c.967-979del13
18	F	HypoT	c.1336C>G/c.967-979del13
19	F	HypoT, HP	c.879 + 1G>A/c.967_979del13
20	M	Autoimmune PAI, HP, Al, CMC	c.1163_1164insA/c.967-979del13
21	F	HP, B12, POI, Al, CMC	c.769C>T/c.769C>T
22	F	Autoimmune PAI, T1D, HP, Al, CMC	c.967-979del13/c.967-979del13

a F, female; M, male.

b Al, alopecia; B12, vitamin B12 deficiency; CMC, chronic mucocutaneous candidiasis; GHdef, growth hormone deficiency; HP, hypoparathyroidism, HyperT, hyperthyroidism; HypoT, hypothyroidism; PAI, primary adrenal insufficiency; POI, primary ovarian insufficiency; T1D, type 1 diabetes; V, vitiligo.

## Discussion

Tregs and their suppressive abilities are of major importance in maintaining peripheral tolerance, and proper Treg function is crucial to avoid autoimmune disease.^48^ These cells have been proposed as a therapy for autoimmune diseases, but standard protocols are not yet available. To establish such protocols, we need to understand how Treg function, expansion, and individual variation affect their behavior. For long it has been advocated that the autoimmune pathology in APS-1 patients is caused by failure of both central and peripheral tolerance as AIRE is essential in thymic expression of tissue-restricted antigens, but also have a yet undissected role in the generation of Tregs.^4^^,^^7^^,^^8^^,^^10^^,^^11^ Notably, a lower level of circulating Tregs is constantly reported in patients with APS-1,^40^^,^^41^^,^^42^^,^^44^ which we here replicated in expanded Tregs with lower expression of CD25. We have previously investigated freshly sorted Tregs by bulk RNA sequencing, with indications of Treg impairment of lipid metabolism and gut homing in APS-1 patients.^45^ Here, we reveal minor transcriptomic and proteomic disturbances of both freshly sorted and expanded Tregs in APS 1 patients, but no significant effect on their polyclonal capacity. Importantly, patients’ Tregs were equally to healthy controls, if not even more, able to expand *in vitro*, with a larger “expansion factor” (52 in patients vs. 37 in controls). There was no evidence of deviant T cell polarization in cells from the APS-1 patients.

As we were already familiar with bulk sequencing patterns of APS-1 Tregs,^45^ we here applied single cell approaches to be able to investigate specific subpopulations that might be deviant. To this end, we first analyzed freshly sorted Tregs from a small number of patients and matched controls without finding large differences between the groups. To increase the sample size, we had to overcome the logistical challenges of simultaneous blood sampling from a relatively large and geographically dispersed APS-1 cohort in Norway, and to obtain enough cells for multiple assays, we had to expand them. This not only increased the cell number, but also allowed us to examine the expansion potential of Tregs in these patients with severe autoimmune phenotypes. Cell expansion might be required for future therapeutic applications of Tregs. After expansion of cells, we verified the Tregs-identity by using specific markers in single cell sequencing, flow and mass cytometry. A few patients showed high levels of NK, Th17 and B cell markers, which could suggest either contamination or polarization of some cells to an intermediate state between Tregs and Th17.^49^ These aberrant cell phenotypes might affect the transcriptome and proteome of the specific samples, but they should not alter the overall analysis. Previous studies have reported reduced FOXP3 levels in Tregs from APS-1 patients.^42^ We only see hints of this in our patients illustrated by lower frequencies of the clusters 2, 5 and 7 containing immune functional genes which could affect Treg function, like *GZMB, TIGIT*, cytokines and partners in interferon stimulation; *FOXP3* did not impact on the clusters. Rather, our study found decreased Treg CD25 protein expression in both flow and mass cytometry on patients. As CD25 is constitutively expressed on Tregs,^50^ we expected the expression to be consistently high in both cohorts. In autoimmune tissue reactions, it is known that FOXP3+CD25^low^ cells can be derived from FOXP3+CD25^high^ T cells and frequencies of FOXP3+CD25^low^ cells can act as markers of peripheral Treg expansion.^51^ How this come to play in cultured Tregs with continuous refill of IL2 is unknown. We propose that APS-1 Tregs may be more prone to proliferation and exhaustion, as indicated by increased levels of MKI67 in the transcriptomic experiments, the higher proteomic level of the exhaustion marker CD57 and data from Laakso et al.^42^ This may impair the ability to suppress autoimmunity caused by AIRE deficiency. Alternatively or additionally, CD25 regulation may be disrupted in APS-1 Tregs, leading to their exhaustion.^52^

Both the expanded and naive Tregs showed some transcriptomic variations between the cohorts, but the log2FC-values were small, and only three genes were differentially expressed in the same direction in the two setups (freshly sorted and expanded cells): *CD52, LTB* (down in patients) and *TXNIP* (up in patients). The lower expression of CD52 might indicate decreased Treg effector capabilities,^53^ and the decrease of LTB supports deficient lipid metabolism, as we also found using bulk sequencing, but then because of altered FASN expression,^45^ as well as lack of Treg stability and deviant migration.^54^^,^^55^ *TXNIP* encodes the thioredoxin-interacting protein, which is an important regulator of glucose metabolism and redox state; the MondoA-TXNIP axis is a critical metabolic regulator of Treg identity and function, and deviations of *TXNIP* expression might therefore impact on Treg overall function.

We found increased HLA-gene expression in expanded Tregs from APS-1 patients, suggesting higher activation and suppression, but this contradicted previous findings in naive T cells.^40^^,^^45^ Protein techniques did not support HLA-differences, implying that the transcriptomic results were due to different HLA-types. Although our overall approach did not show other large differences between APS-1 and healthy controls, several functional Treg markers were found to be downregulated in patients in the expanded cells’ transcriptomic profiling; 1) Functional Treg molecules included in cluster 2,5 and 7; 2) Chemokines and their receptors; 3) Granzyme *GZMB* and regulation of killing activity. However, the whole profile is complex and does not unilaterally support functional defects of Tregs. Granzymes are thought to be important for the suppressive capacity of Tregs,^56^^,^^57^^,^^58^ and downregulation does indicate a lower function of Tregs. However, differences are small and the relevance for biological function unclear. The constant downregulation of CCL3, 4 and 5 in expanded Tregs of APS-1 patients might indicate contamination of NK cells, but can also mean disturbances of Treg trafficking, but this was not verified on freshly sorted Tregs. Chemokines and chemokine receptors should be dramatically decreased when culturing Tregs for expansion^59^ due to the lack of trafficking opportunities for cells *ex* vivo. Hence, the mechanistic role for the downregulation of chemokines in patients remain uncertain.

From the proteomic approaches, several markers involved in Treg function were differentially expressed between patients and controls in expanded Tregs. Lower levels of CD31, CD103 and Helios concomitant with higher levels of the exhaustion marker CD57 all indicate less biological functionality by different mechanisms,^60^^,^^61^^,^^62^^,^^63^ while upregulation of CD161 suggests a suppressive phenotype also capable of producing proinflammatory mediators.^64^ While CD31 and CD103 are expressed in very low amounts and might not be very important for the function of expanded Tregs, there are quite large differences in the more highly expressed Helios, CD57 and CD161. How this can be interpreted in the context of expanded cells is not completely clear.

As the cell profiling experiments hinted at subtle, but confusing, functional impact of *AIRE* mutations for Treg function, we wanted to analyze the *in vitro* functionality of Tregs. The established flow cytometry–based suppression assay revealed no differences between APS-1 patients and matched controls, and some patients had even slightly higher suppressive capacity. This contrasts with Kekäläinen et al. who reported on numeral and functional perturbations of APS-1 patients’ Tregs, supported by a lower suppressive capacity.^41^ However, their study used non-expanded cells, while we applied more stringent Treg isolation and a more sensitive flow cytometry protocol. We believe that these methodological improvements explain the discrepancy. Although we acknowledge the difference between freshly sorted and expanded Tregs, our data provide evidence that polyclonal Treg responses in APS-1 patients are intact. Detailed knowledge on the TCR repertoire of Tregs may furthermore indicate whether deviations regarding target-specific reactivity are present. Indeed, we speculate that APS-1 patients lack AIRE-dependent Treg specificities necessary for avoiding development of autoimmune disease, in concordance with previous *Aire*^*−/−*^ mice studies, where it has been shown that a missing or non-functional *Aire* drives cells with Treg-biased clones into autoreactive T cells.^11^ This is in agreement with Sng et al., who reported that some clones with TCRDV rearrangements normally present in the Treg pool was converted into CD4^+^ T effector cells.^40^ However, we here show a restricted TCR repertoire of Tregs only for some APS-1 patients, which could interfere with antigen-specific suppressive responses.^11^ Overall, patients and healthy controls did not differ significantly from each other regarding the expanded Treg immune repertoire.

Although our analyses of freshly sorted and expanded Tregs are not complete, they shed light on the potential of using *in vitro* expanded Tregs to repair tolerance imbalance in APS-1. *In vitro* expanded Tregs have previously been shown to significantly inhibit CD8+ T cell proliferation, and the transcriptome of Tregs isolated from PBMC and of expanded Tregs are interlaced,^65^ showing that expanded Tregs indeed have similar functions as non-expanded Tregs. Several articles have further investigated properties of expanded Tregs from healthy persons and from immune disorders, including Crohn’s disease, and found that Tregs expanded *ex vivo* retain stable FOXP3 expression and even increase their suppressive capacity.^66^^,^^67^ Jarvis et al. recently showed that expanded human Tregs switch their metabolism to aerobic glycolysis and that the suppressive function is driven by CD39 and CD73 expression, emphasizing that these cofactors must be controlled, or even enhanced, in expansion protocols.^68^

In conclusion, we present a comprehensive study on single-cell proteomic and transcriptomic analyses on freshly sorted and expanded Tregs from APS-1 patients. We found that AIRE deficiency had little effect on the transcriptome of freshly sorted Tregs, but increased their expansion potential, resulting in functional but slightly exhausted cells. Expanded Tregs from APS-1 patients suppressed autologous responder cells as well as those from controls, but some had a narrower TCR repertoire. Our findings do not support APS-1 as a functional Tregopathy, but rather that inadequate generation of Tregs in the thymus or periphery contributes to a decreased overall number of Tregs. The expandability of APS-1 Tregs implies that they could be used for *in vitro* manipulation and therapy, but their functionality needs further evaluation.

### Limitations of the study

We based our hypotheses on Aire deficient mice studies that showed thymic Treg defects.^37^^,^^39^ The thymus is inaccessible in the patients and informative studies would probably require younger patients. Therefore, we needed to perform our studies on blood, where Tregs constitute about five to 7% of CD4^+^ T cells. Our choice to utilize *in vitro* expanded Tregs for downstream analyses enables multiple molecular studies to describe the functionality of APS-1 patients’ Tregs in an expanded state. On the downside, this strategy may affect the phenotype and stability of Tregs, as a previous study showed loss *FOXP3* expression.^69^ Therefore, we cannot directly extrapolate our findings to *in vivo* Tregs or Treg therapy. We also caution against comparing freshly sorted and expanded Tregs, as they differ in origin, state, and activation. For the single cell transcriptomics, we used each cell as a replicate instead of each individual. This may artificially increase the n and decrease the p value and inflate the number of differentially expressed genes with low fold changes.^70^

Two patient samples did not go through the “reads-mapped-to-any-V(D)J-gene” quality control. These were discarded from further analysis, but we cannot differentiate whether this bad QC-measure reflects technical or biological issues with the samples. Hence, we might have failed to include two informative patient samples with very restricted TCR repertoires.

We furthermore acknowledge the limitation of studying polyclonal rather than antigen specific Tregs. Indeed, successful epitope mapping of CD8+ responses against the main adrenal autoantigen for adrenal failure and APS-1, 21-hydroxylase, has been reported by us and others,^71^^,^^72^ but there is no successful protocol for APS-1-relevant CD4^+^ autoantigen responses.

## STAR★Methods

### Key resources table

**Table:** 

REAGENT or RESOURCE	SOURCE	IDENTIFIER
**Biological samples**		

Patient naïve Tregs	Local hospitals	
Patient expanded Tregs	Local hospitals	

**Critical commercial assays**		

Chromium Next GEM Single Cell 3’ GEM, Library and Gel Bead Kit v3.1	10X Genomics	1000128
Chromium Next GEM Chip G Single Cell Kit	10X Genomics	1000127
MACSxpress Whole Blood Treg Isolation Kit Human	Miltenyi Biotec	130-109-557
Treg Expansion Kit Human	Miltenyi Biotec	130-095-345
Next GEM Single Cell 5’ Reagent Kit v2 (Dual Index)	10X Genomics	1000265
Chromium Next GEM Chip K Single Cell Kit	10X Genomics	1000286
TCR Amplification Kit	10X Genomics	1000252
Library Construction Kit	10X Genomics	1000190
Human Immunology Panel	10X Genomics	1000259
Target Hybridization Kit	10X Genomics	1000248
PAN T cell Isolation Kit Human	Miltenyi Biotec	130-096-535

**Antibodies**		

CD3	BD	561416; RRID: AB_10612021
CD4	BioLegend	300530; RRID: AB_893328
CD25	BD	335824; RRID: AB_2868687
CD45RA	BD	560674; RRID: AB_172749
CTLA4	BioLegend	369606; RRID: AB_2616795
CD39	Thermo Fisher Scientific	12-0399-42; RRID: AB_1272091
CD31	BD	744757; RRID: AB_2742460
HLA-DR	BD	564231; RRID: AB_2738685
CD8	BD	555368; RRID: AB_395771
FoxP3	BD	563955; RRID: AB_2738507
Helios	BioLegend	137222; RRID: AB_10662535
CD3	BioLegend	300440; RRID: AB_314060
CD25	BioLegend	302606; RRID: AB_314275
CD56	BioLegend	362516; RRID: AB_2564088
CD14	BioLegend	301864; RRID: AB_2860767
CD127	BioLegend	351310; RRID: AB_10960140
CD8a	BioLegend	301040; RRID: AB_2563185
CD21	BioLegend	354905; RRID: AB_2561453
CD4	BioLegend	317426; RRID: AB_571942
CD19	Miltenyi Biotec	130-091-248; RRID: AB_244221
CellTrace Violet Cell Proliferation Kit	Thermo Fisher Scientific	C34557
Live/dead Fixable Yellow Dead Cell Stain kit	Thermo Fisher Scientific	L34959
CD103	Fluidigm	3151011B; RRID: AB_2756418
CD123 (IL3R)	Fluidigm	3143014B; RRID: AB_2811081
CD127 (IL7Ra)	Fluidigm	3149011B; RRID: AB_2661792
CD14	Invitrogen	11514562; RRID: AB_1071260
CD152 (CTLA4)	Fluidigm	3161004B; RRID: AB_2687649
CD161	Fluidigm	3164009B; RRID: AB_268765
CD19	Fluidigm	3142001B; RRID: AB_2651155
CD25 (IL2Ra)	Fluidigm	3169003B; RRID: AB_2938861
CD27	Fluidigm	3158010B; RRID: AB_2858231
CD274 (PD-L1)	Fluidigm	3159029B; RRID: AB_2861413
CD278 (ICOS)	Fluidigm	3148021D; RRID: AB_281106
CD279 (PD1)	Fluidigm	3155009B; RRID: AB_2811087
CD28	Fluidigm	3160003B; RRID: AB_2868400
CD3	Fluidigm	3170001B; RRID: AB_2811085
CD31/PECAM1	Fluidigm	3144023B; RRID: AB_3096014
CD4	Fluidigm	3145001B; RRID: AB_2661789
CD45	Fluidigm	3089003B; RRID: AB_2938863
CD45RA	Fluidigm	3153001B; RRID: AB_2802108
CD45RO	Fluidigm	3165011B; RRID: AB_2756423
CD5	eBioscience	14005982; RRID: AB_467083
CD56 (NCAM)	Fluidigm	3163007B; RRID: AB_3096015
CD57	Fluidigm	3176019B; RRID: AB_2858249
CD66b	Novus Biologicals	NBP2-80664; RRID: AB_3096017
CD69	Fluidigm	3162001B; RRID: AB_3096016
CD8	Fluidigm	3168002B; RRID: AB_2892771
HLA-DR	Fluidigm	3174001B; RRID: AB_2665397
Tigit	Fluidigm	3154016B; RRID: AB_2888926

**Oligonucleotides**		

β-actin_Fw	Sigma-Aldrich	5’- GCATGGGTCAGAAGGATTCCT
β-actin_Rv	Sigma-Aldrich	5’- TCGTCCCAGTTGGTGACGAT
CCR4_Fw	Eurogentec	5’- CTGTATTCCTTGGTTTTTGT
CCR4_Rv	Eurogentec	5’- AGGTCCTTGCCCTCAAGGA
CXCR3_Fw	Eurogentec	5’- CTACACCGAGGAAATGGG
CXCR3_Rv	Eurogentec	5’- TGCAACTGCCCAGAAGGGA
FOXP3_Fw	Eurogentec	5’- ATGCACCAGCTCTCAA
FOXP3_Rv	Eurogentec	5’- AGTGGGTAGGAGCTCT
GATA3_Fw	Sigma-Aldrich	5’- TCATTAAGCCCAAGCGAAGG
GATA3_Rv	Sigma-Aldrich	5’- GTCCCCATTGGCATTCCTC
ROR γ t_Fw	Sigma-Aldrich	5’- TGGACCACCCCCTGCTGAGAAGG
ROR γ t_Rv	Sigma-Aldrich	5’-CTTCAATTTGTGTTCTCATGACT
Tbet_Fw	Sigma-Aldrich	5’- GATGCGCCAGGAAGTTTCAT
Tbet_Rv	Sigma-Aldrich	5’- GCACAATCATCTGGGTCACATT

**Deposited data**		

Single-cell RNA seq data	This paper	Gene Expression Omnibus (GEO) reference number GSE243061 and European Genotype-Phenome Archive (EGA) reference number EGAD50000000260, reference number EGAD50000000261, reference number EGAD50000000262 project number EGAS50000000181
Flow- and mass cytometry data	This paper	Mendeley data- https://doi.org/10.17632/72hvtcwktb.1 (flow cytometry) or https://doi.org/10.17632/bxv78zszvc.1 (mass cytometry).

**Software and algorithms**		

GraphPad Prism 9	Dotmatics	Home - GraphPad
FlowJo v10.2 and v10.8 CL	BD	Home | FlowJo, LLC
R v4.2.0	Cran, The R Foundation	R: The R Project for Statistical Computing (r-project.org)
Cell Ranger suite v.6.1.2	10X Genomics	Installing Cell Ranger -Software -Single Cell Gene Expression -Official 10x Genomics Support
Seurat (v4.1.0, R package)	Hao et al.^73^	GitHub - satijalab/seurat: R toolkit for single cell genomics Tools for Single Cell Genomics ⋅ Seurat (satijalab.org)
Biorender	Biorender.com	Biorender.com

### Resource availability

#### Lead contact

Further information and requests for resources and reagents can be directed to and will be fulfilled by the lead contact, Anette S. B. Wolff (Anette.Boe@uib.no).

#### Materials availability

This study did not generate new unique reagents.

#### Data and code availability

• The raw transcriptomics data of blood Tregs from Finnish patients has been deposited in GEO (Gene Expression Omnibus) with accession number GSE243061. The transcriptomic data of expanded Tregs from Norwegian patients has been submitted to the European Genotype-Phenome Archive (EGA) via the Norwegian Federated EGA (reference numbers EGAD50000000260, EGAD500000002601 and EGAD50000000262 and project number EGAS50000000181 (https://ega-archive.org/studies/EGAS50000000181)). Summarized data is additionally available in the amended Tables S5, S6, S7, and S8.
• Flow and mass cytometry data are available from “Mendeley data” with the accession numbers https://doi.org/10.17632/72hvtcwktb.1 (flow cytometry) og https://doi.org/10.17632/bxv78zszvc.1 (mass cytometry).
• Methodology for the bioinformatics is provided in full under “method details”; “bioinformatics analysis of freshly sorted Tregs” and “alternative strategy for bioinformatics analysis for expanded Tregs”. It is also given in the key resources table.

### Experimental model and study participant details

#### Patients and controls

We included four APS-1 patients (all females, mean age 51.5 (range 36-66) years) and four age and gender matched healthy controls (all females, mean age 48.3 (range 30-63) years) from Finland to perform single-cell sequencing on freshly sorted Tregs (Table 1 #1-4). This represents a small cohort of APS-1 Tregs, and was performed in Tartu, Estonia, with research permission HUS/1127/2016, including ethical review board approval from Helsinki University Hospital (HUS) Medical Ethical Review Board.

Eighteen APS-1 patients (nine females, nine males, mean age 45.8 (range 20-71) years) enrolled in the Norwegian Registry for Organ-Specific Autoimmune Disorders (ROAS) were included for the expanded Tregs-part (Table 1 #5-22). Whole blood from 20 sex- and age-matched healthy donors (eleven females, nine males, mean age 44.6 (range 20-70) years) was obtained from the Blood Bank at Haukeland University Hospital (Bergen, Norway). All donors included in this research project was of Caucasian heritage. No socioeconomic information was available. Detailed data on gender and usage of patient and healthy control material in the applied experiments is found in Table S9. This study was approved by the Regional Committee for Medical and Health Research Ethics, Norway (Study no. 2018/1417, 2013/1504 and 2009/2555). All patients signed a consent form to participate in the project and healthy controls gave their informed consent for research when donating blood, in accordance with the Declaration of Helsinki.

### Method details

#### Protocol for single-cell transcriptomics of sorted Tregs from blood cells

PBMCs from four patients and four controls (Table 1, #1-4) were thawed slowly using RPMI 1640 media (Corning) supplemented with Penicillin-Streptomycin (Corning, cat. 30-002-CI) and Fetal Bovine Serum (Gibco, cat. 10270106). After washing with media, cells were stained with antibodies against CD3, CD25, CD14, CD56, CD127, CD8a, CD21, CD4 and CD19 (Table S1A). Tregs were sorted out as CD4^+^CD25^+^ CD127^low^ cells using a Sony MA900 Cell Sorter (Sony Biotechnology, USA) and counted using Luna FL Automated Cell Counter and Acridine Orange/ Propidium Iodide Stain (Logos Biosystems). Two thousand carefully counted Tregs were loaded onto a Chromium controller and cDNA was generated using Chromium Next GEM Single Cell 3′ GEM, Library & Gel Bead Kit v3.1 (10X Genomics, cat. 1000128) and Chromium Next GEM Chip G Single Cell Kit (10X Genomics, cat. 1000127) according to the manufacturer’s instructions.

#### Treg isolation and *in vitro* expansion

CD4^+^CD25^+^CD127^low^ cells (from here-on referred to as “expanded Tregs”) were isolated directly from EDTA-blood using the MACSxpress Whole blood Treg Isolation Kit Human (Miltenyi Biotec, cat. 130-109-557). The purity of up-concentrated Tregs was not investigated because we needed all cells for the downstream protocol but has been evaluated to contain >65% FOXP3+ Tregs in similar experiments.^45^ Cells were then expanded in TexMACS Medium (Miltenyi Biotec, cat. 130-097-196) supplemented with 500 U/mL recombinant (r) - IL2 (Miltenyi Biotec, cat. 130-097-744) and 5% FBS or human AB serum for 14 days at 37°C and 5% CO_2_. At day 0, 2x10^7^ CD3/CD28 MACSiBead Particles/mL were added, according to the Treg Expansion Kit Human protocol (Miltenyi Biotec, cat. 130-095-345). Cells were given new medium containing 500 U/mL rIL2 every 2-3 days and split when necessary. At day 14 cells were harvested, counted and frozen in AB serum or FBS supplemented with 10% DMSO.

#### 10X single-cell sequencing of *in vitro* expanded Tregs

*In vitro* expanded Tregs from nine APS-1 patients (Table 1, #5-13) and nine healthy controls were thawed and dead cells removed with the Dead cell removal kit (Miltenyi Biotec, cat. 130-090-101). Cells were carefully counted, pelleted and dissolved to 1x10^6^ cells/ml in PBS supplemented with 0.5% bovine serum albumin (BSA). Ten thousand cells were loaded onto the Chromium controller, and cDNA was generated from RNA captured on beads, with 11 PCR cycles, all with the Next GEM Single Cell 5’ Reagent Kit v2 (Dual Index) (10X Genomics, cat. 1000265) and the Chromium Next GEM chip K Single Cell Kit (10X Genomics, cat. 1000286), according to the manufacturer’s protocol (Rev A, 10X Genomics). Gene expression (GEX) and TCR library construction was also carried out according to the manufacturer’s protocol (Rev A, 10X Genomics) using the TCR Amplification Kit (10X Genomics, cat. 1000252) and the Library Construction Kit (10X Genomics, cat. 1000190).

For the targeted Human Immunology Panel (10X Genomics, cat. 1000259) (Table 1, #5-12), eight and eight GEX libraries were pooled (four patients and four controls per pool) and input was based on concentrations of the GEX libraries for each sample, ranging from 75.2 to 164.6 nM. The SPRIselect Library Concentration for Targeted Gene Expression Protocol (Rev D, 10X Genomics) and the Target Hybridization Kit (10X Genomics, cat. 1000248) were used to concentrate GEX libraries. For the Human Immunology Panel library amplification, 12 cycles were used on the thermal cycler. All quality controls and quantifications were performed using the Agilent High Sensitivity D5000 ScreenTape System and the Agilent TapeStation 4200 Instrument (Agilent Technologies, cat. 5067-5593, 5067-5592, 401428 and 401425).

Final libraries were quality checked on Agilent TapeStation 4200 (Agilent Technologies) and quantified by qPCR using the KAPA Library Quantification Kit. Libraries were pooled in equimolar amounts and paired-end sequenced on an Illumina NovaSeq 6000 instrument on a NovaSeq SP flow cell. Parameters used for sequencing were 26x10x10x90 base pairs and targeted number of reads per cell were 7000 for the TCR libraries and 3000 for the Immune Panel.

#### Bioinformatics analysis of freshly sorted Tregs

The raw sequencing data were processed using the 10X Genomics Cell Ranger pipeline v.6.1.2 with the GRCh38 reference. The scRNA-seq alignment and quantification were carried out on the Tartu University High-Performance Computing Center Rocket cluster. The low-quality cells were removed based on the UMI and gene count distributions in each sample. The transcriptomes of the retained high-quality cells were further processed according to the Seurat data integration workflow to account for the between-batch technical differences as previously described.^74^ In brief, the data were normalized using the SCTransform function v.2. The integration features were selected as the intersection of the top highly variable genes. Next, the iterative pairwise integration was performed for all the samples. After the integration, the PCA was performed, and the first 30 principal components were selected for building the UMAP projection and clustering the data. Pathway analysis was performed with Ingenuity on globally differentially expressed genes in an attempt to define the different clusters.

#### Alternative strategy for bioinformatics analysis for expanded Tregs

Raw BCL files were demultiplexed with the mkfastq pipeline of the Cellranger suite v.6.1.2, implemented and curated by 10X Genomics. The quality of the resulting FASTQ files was assessed by inspecting the results of the fastqc v.0.11.6 screening, performed on each sample to detect possible issues in the library preparation and sequencing phases. Cellranger count pipeline from the 10X Cellranger suite was used for alignment to the human reference genome (build GRCh38) and for counting reads associated to each feature. As a result, a feature-barcode matrix was produced for each sample. The results of the alignment were quality controlled by analysing the alignment statistics. The function emptyDrops from DropletUtils v.3.15 was applied on the raw (unfiltered) features-barcodes matrices to estimate the presence of possible empty droplets.

Scanpy v.1.9.1 was run in a per sample basis to operate the quality filtering. In particular, the following criteria were applied: any droplet with an FDR from emptyDrops > 0.005, any feature not found in at least 1 cell, any cell with more than 15% of counts associated with mitochondrial genes and any cell with less than 10 genes by count were all removed from further analysis. Doublet detecting was then performed using Solo implemented in the scVI v.0.6.8 package. Solo was run on a lane-by-lane basis, as each lane will have different technical variation.

All the patient and control samples were processed relying on a customized workflow scripted in the R v.4.2.0 environment, based on Seurat v.4.1.0 suite.^73^ In particular, the relevant features of the data related to each sample were first analysed summarized in a Seurat object and, subsequently, the objects related to each sample class (e.g. patients and controls) were integrated (Seurat:IntegrateData) to produce “Integrated patient” and “Integrated control” objects, respectively. The Integrated Objects, summarised in a single entity of the relevant features of the composing samples, were used for the subsequent analysis: (1) identification of cellular subpopulations (clustering) and characterisation of markers and (2) differential marker (gene) expression. These analysis steps were performed considering and comparing the immune panel composing the patient and control samples.

The raw BCL files for TCR analysis and VDJ clonotypes were demultiplexed relying on the mkfastq pipeline of the Cellranger suite v.6.1.2, implemented and curated by 10X Genomics. The quality of the resulting FASTQ files was assessed by inspecting the results of the fastqc v.0.11.6 screening, performed on each sample to detect possible issues in the library preparation and sequencing phases. Clonotypes were defined as the combined TRA+TRB amino acid sequences. The alignment of the FASTQ files to the references and subsequent quantification of the clonotypes was performed relying on the Cellranger VDJ pipeline. Plots and figures were produced starting from the .vloupe files obtained from the Cellranger VDJ pipeline. Plots and figures were produced with custom scripts in the R environment, based mainly on the ggplot2 v.3.3.6 and Seurat suites. Additional figures were made using the GraphPad Prism 9.1.0 software (GraphPad Software). Unsupervised clustering was used to identify the different clusters, using marker genes. The most upregulated genes in both cohorts in each cluster was loaded onto String (https://string-db.org/)^75^ and gene ontology and pathway analysis (Ingenuity) was performed in an attempt to define the different clusters.

#### Calculation of TCR repertoire diversity

The Shannon Diversity Index^76^ for clonotypes ≥0 was calculated for each individual patient and each control, defined as $Shannon\;Diversity\;Index=-\sum_{s}f\left(s\right)\cdot \log\;f\left(s\right)$. Further, $f\left(s\right)=\frac{n}{N}$ where n equals a specific clone and N equals the total number of clones. This means that for each individual, the total number of TCR clonotypes was rarified. The Shannon Diversity Index was calculated using Microsoft Excel 2016.

#### Flow cytometric analysis to characterise *in vitro* expanded Tregs

Expanded Tregs isolated from whole blood of 17 APS-1 patients (Table 1, #5-10 and #13-22) and 14 healthy controls were characterized by flow cytometry. Cells were stained with a modified protocol according to Santegoets et al.^45^^,^^77^ (Table S1A). Fixation and permeabilization was achieved using the Foxp3/Transcription Factor Staining Buffer Set (eBioscience) according to instructions from the manufacturer. Cells were analysed using the BD LSRFortessa Cell Analyser and the BD FACSDiva Software. FlowJo v10.2 and v10.8 CL (BD) was used to analyse flow cytometric data.

#### Expanded Treg characterization by time-of-flight mass cytometry (CyTOF)

Thawed expanded Tregs from 17 APS-1 patients (Table 1, #5-10 and 13-22) and 17 healthy controls were barcoded according to the Cell-ID 20-Plex Pd Barcoding Kit User Guide (Fluidigm, cat. 201060). Before antibody labelling, cells were incubated for 10 minutes at room temperature with 1 μ L Fc-block solution (BD) and 1 μ L heparin (10U) (Sigma, cat. H3393). A cocktail of 27 metal-conjugated antibodies (Table S1B) was added and incubated at room temperature for 30 minutes. CD14, CD5 and CD66b were conjugated in-house using the Fluidigm Antibody Labelling Kit (Fluidigm, cat. 201112A, 201166B and 201141A). After washing, an intercalation solution (700 μ L PBS + 250 μ L fresh 16% PFA (Thermo Scientific, cat. J19943.K2) + 100 μ L 10X Intracellular Staining Perm Wash Buffer (BioLegend, cat. 421002) + 0.25 (125nM) μ L 500 μ M Iridium Intercalator (Fluidigm, cat. 201192B)) was added and incubated at 4°C overnight. Cells were washed, pelleted and frozen in CRYO#20 (Cytodelics, cat. hC002-1000) at -80°C until acquisition on the CyTOF XT instrument (Fluidigm).

Samples were debarcoded using the Fluidigm Debarcoding Software (7.0.8493.0) with a 20-plex-debarcoding key (Fluidigm). Raw CyTOF XT FCS files were bulk normalized in the fluidic CyTOF Software v8.0 using EQ four-element calibration bead (bead normalization passport) to normalize the data sets. Randomization was selected automatically on the uniform negative distribution (UND) in linear value, compatible with FlowJo v10.2 (BD), and on the default time interval normalization. The normalized FCS files were exported to RStudio 2022.12.0+353 for concatenation and debarcoding using CATALYST 1.22.0.^78^ The Premessa R package v0.3.4^79^ was aligned with all the debarcoded individual FCS files containing unique samples to fix channel names and for removing background channels. For panel discrepancies, renamed FCS files were thoroughly checked in Cytobank. The data cleanup strategy was adopted as standard on event_length, 140-bead channel, and Ir191/Ir193 versus time, followed by the four Gaussian parameters (Center, Offset, Width, and Residual). The CD45^+^ population, representing nucleated hematopoietic cells along with its precursors, of the FCS files (including standard internal control) from four batches were downloaded from Cytobank and imported into RStudio 2022.12.0+353 for batch correction using Cydar with ncdfFlow v.2.36.0^80^ and flowCore v.2.2.0^81^ as dependency packages.^82^^,^^83^ Twenty-seven channels were scaled on a cofactor of 5 using arcsinh transformation.^84^ Samples were analysed using FlowJo v10.2 CL (BD), using linear axes after batch correction.

#### qPCR of expanded Tregs

Expanded Tregs were washed with PBS and pellets were resuspended, run through an RNA QIAshredder column (Qiagen # 800000008015) and frozen immediately at -80°C in 350 μl of RLT lysis buffer from the RNeasy Mini Kit (Qiagen #74104). The RNeasy Mini Kit was further followed according to the manufacturer’s protocol for RNA isolation, and cDNA was prepared using the Superscript IV VILO Kit with EZ DNase enzyme (Invitrogen, Thermo Fisher Scientific # 11766050) in accordance with the instructions provided by the kit with 400 ng of input RNA. Generated cDNA was diluted 20 times before being used in SYBR Green qPCRs.

Expression of the genes *FOXP3*, *RORγt, GATA3*, *Tbet, CCR4* and *CXCR3* were evaluated using 2x PowerTrack SYBR Green kit using standard protocols with gene specific primers (5% v/v from 1:10 dilution each) (Table S2). *Beta-actin* was used as housekeeping gene. The samples were run and analysed by the QuantStudio 5 Real-time PCR system. All samples were tested in triplicates. The 2^–ΔΔCt^ method was used to calculate calibrated expression levels based on beta-actin expression. The calibrator for fold change values was the mean of healthy controls.

#### Peripheral blood mononuclear cell (PBMC) isolation

PBMCs were isolated from APS-1 patients and healthy controls using Ficoll density gradient centrifugation using standard protocols. Cells were frozen in human AB serum or fetal bovine serum (FBS) supplemented with 10% dimethyl sulfoxide (DMSO) and stored at -150°C until use.

#### Co-culture suppression assay

Thawed PBMCs from 15 APS-1 patients (Table 1, #1-15) and 15 healthy controls were run through the Pan T Cell Isolation Kit Human (Miltenyi Biotec, cat. 130-096-535) and were used to obtain responder T cells (Tresp), according to the manufacturer’s protocol. Cells were then cultured in TexMACS Medium supplemented with 5% AB serum or FBS and 50 U/ml rIL2, to a concentration of 1x10^6^ cells/ml. Cells were rested overnight at 37°C and 5% CO_2_. Next day, Tresp cells were stained with the CellTrace Violet Cell Proliferation Kit (Invitrogen, cat. C34557) for flow cytometry according to instructions from the manufacturer. Cells were dissolved to a concentration of 5x10^5^ cells/ml in TexMACS Medium supplemented with 5% AB serum or FBS, 1% penicillin-streptomycin and 50 U/ml rIL2, further referred to as Treg suppression medium.

To assess the suppressive capacity, recovered expanded Tregs were activated with 3 μ L/mL cells Immunocult Human CD3/CD28 T Cell Activator (Stemcell Technologies, cat. 10971) and co-cultured at different ratios (Tresp:Tregs 1:1, 2:1, 4:1 and 8:1) in Treg suppression medium for 5 days at 37°C and 5% CO_2_. Expanded Tregs from patients were added to autologous patient responder cells and expanded Tregs from healthy controls were added to autologous responder cells from healthy controls. Cells were harvested and stained with Live/Dead Fixable Yellow Dead Cell Stain Kit (Invitrogen), and directly conjugated mouse anti-human antibodies against CD3, CD4, CD8 and CD25 (Table S1). Cells were fixed using the Foxp3/Transcription Factor Staining Buffer Set (eBioscience, cat. 00-5523-00) according to instructions from the manufacturer. Suppressive capacity was assessed using flow cytometry (BD LSRFortessa Cell Analyser and the BD FACSDiva Software). Percentage of Treg suppression was calculated as

$$\%\;Treg\;suppression=\left(\frac{{Tresp}_{alone}-{Tresp}_{treated\;with\;Tregs}}{{Tresp}_{alone}}\right)\cdot 100\%$$

FlowJo v10.2 and v10.8 CL (BD) was used to analyse flow cytometric data.

#### Enzyme-linked immunosorbent assay (ELISA)

The Quantikine HS ELISA Human IL10 (RnD Systems, cat. HS100C), Human TGF beta 1 ELISA kit (Abcam, cat. ab100647) and Human Interleukin 35 (IL35) ELISA Kit (Nordic BioSite, cat. EKX-6FHVKH-96) were used to measure the amount of Treg-specific cytokines in Treg suppression assay supernatant, according to instructions from the manufacturers. For IL35, samples were diluted 1:1 in sample dilution buffer, for IL10 and TGF-β, samples were handled according to manufacturer’s protocols. Absorbance was read at A450 nm (IL35 and TGF-β), and A490 nm (IL10) using the SpectraMax plus 384 Microplate Spectrophotometer and SoftMax Pro 7.1 software (Molecular Devices).

#### Statistical analysis and figures

An unpaired, parametric t-test was used to examine differences between APS-1 patients and healthy controls for ELISA, Treg characterization by flow cytometry, CyTOF and for Treg suppression. For the Shannon Diversity Index and QPCR, a Mann-Whitney test was used to compare patients and controls. A p-value less than 0.05 was considered statistically significant. All statistical analyses were performed and figures made using GraphPad Prism 9.1.0 if not stated otherwise.

Histograms for each patient’s contribution to the clusters and pai charts for relative cell counts in the ten clusters for expanded Tregs were performed with Graphpad Prism v.10. Statistics were done in the same program using a Mann-Whitney t-test with a significance threshold level of adjusted p<0.05.

Standard deviations are added to all bars in the graphs.

## Supplemental information

Document S1. Figures S1–S21 and Tables S1–S4 and S9

Table S5. Differentially expressed genes in APS-1 patients vs. healthy controls (adjusted p < 3e-14) from freshly sorted Tregs (4 APS-1 patients, 4 healthy controls), related to main Figure 1 APS1 autoimmune polyendocrine syndrome type 1 patients; HC healthy control subjects; p_adj adjusted p value; FC fold change.

Table S6. The most upregulated genes in the ten clusters from expanded Tregs in 8 APS-1 patients and 8 healthy controls, related to main Figure 2 pct_1 percentage positive cells in this cluster; pct_2 percentage positive cells in all other clusters; p_adj adjusted p value; FC fold change). Only genes that are significantly differentially expressed between the clusters (adjusted p < 0.05) and with log2FC > 2 are included.

Table S7. The 20 most differentially expressed genes between APS-1 patients (N = 8) and healthy controls (N = 8) in the ten clusters for expanded Tregs, related to main Figure 2 pct_1 frequency APS-1 cells; pct_2 frequency healthy control cells (p_adj adjusted p value; FC fold change). Only genes that are significantly differentially expressed between the groups (adjusted p < 0.05) and with the largest +/− log2FC > 2 are included.

Table S8. Globally differentially expressed genes in expanded Tregs from 8 APS-patients vs. 8 healthy controls (adjusted p < 0.001), related to main Figure 2 APS1 autoimmune polyendocrine syndrome type 1 patients; HC healthy control subjects; p_adj adjusted p value; FC fold change).
