# Supplementary material for: Single cell characterization of blood and expanded regulatory T cells in autoimmune polyendocrine syndrome type 1
Source: iScience. 2024 Mar 27;27(4):109610. doi: 10.1016/j.isci.2024.109610 (PMC11022049; doi:10.1016/j.isci.2024.109610)
Supplement: Document S1. Figures S1–S21 and Tables S1–S4 and S9 [file mmc1.pdf]

iScience, Volume *27*

Supplemental information

Single cell characterization of blood and expanded regulatory T cells in autoimmune polyendocrine syndrome type 1

Thea Sjøgren, Shahinul Islam, Igor Filippov, Adrianna Jebrzycka, André Sulen, Lars E. Breivik, Alexander Hellesen, Anders P. Jørgensen, Kari Lima, Liina Tserel, Kai Kisand, Pärt Peterson, Annamari Ranki, Eystein S. Husebye, Bergithe E. Oftedal, and Anette S.B. Wolff

Figure S1. Pathway and target network analysis of freshly sorted Tregs in APS-1 patients (N=4) and healthy controls (N=4). ***Related to main Figure 1.***

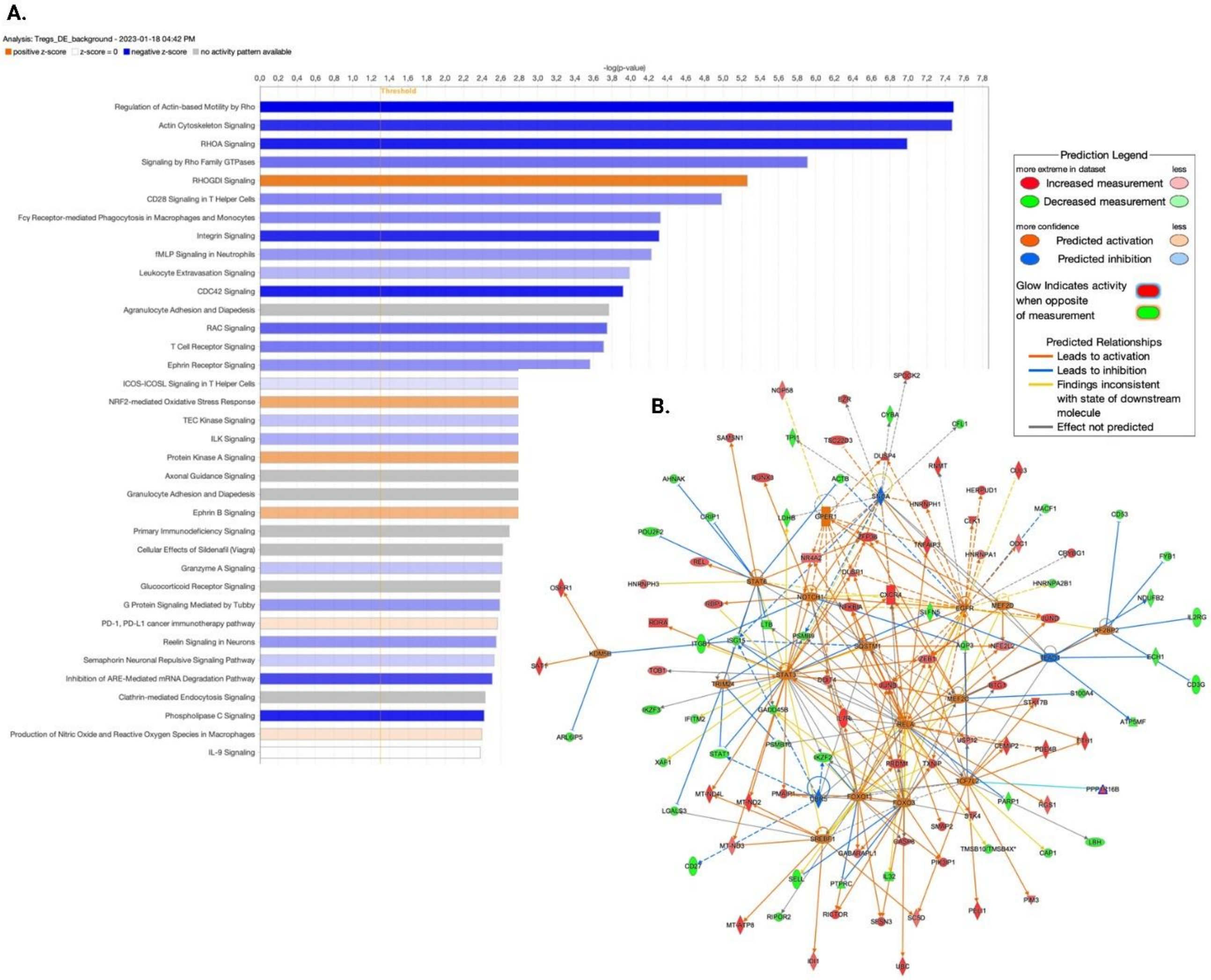

(A) Ingenuity pathway analysis of significant differentiated genes of freshly sorted cells between APS-I patients and healthy controls. (B) Target network analysis based on measured local fusion centres (LFC).

Figure S2. Flow cytometry expression of FOXP3 after the expansion of Tregs. ***Related to main Figure 2.***

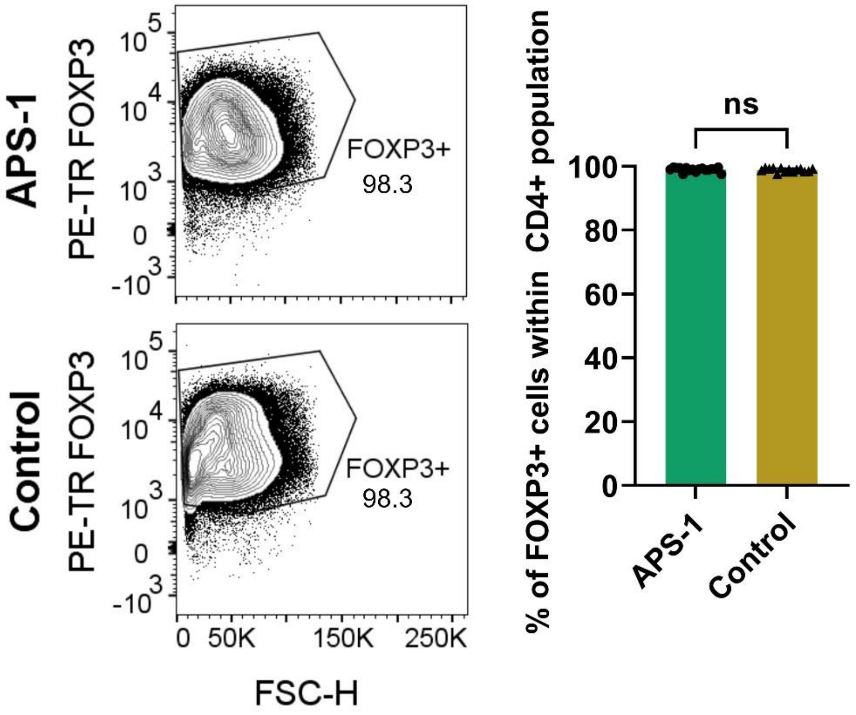

Flow figures are shown for a representative APS-1 patient and a healthy control. FOXP3 expression is shown within the CD4+ population. The P value was calculated using an unpaired, parametric t-test. ns; non-significant.

Figure S3. qPCR on RNA from expanded Tregs in APS-1 patients (N=9) and healthy controls (N=9). ***Related to main Figure 2.***

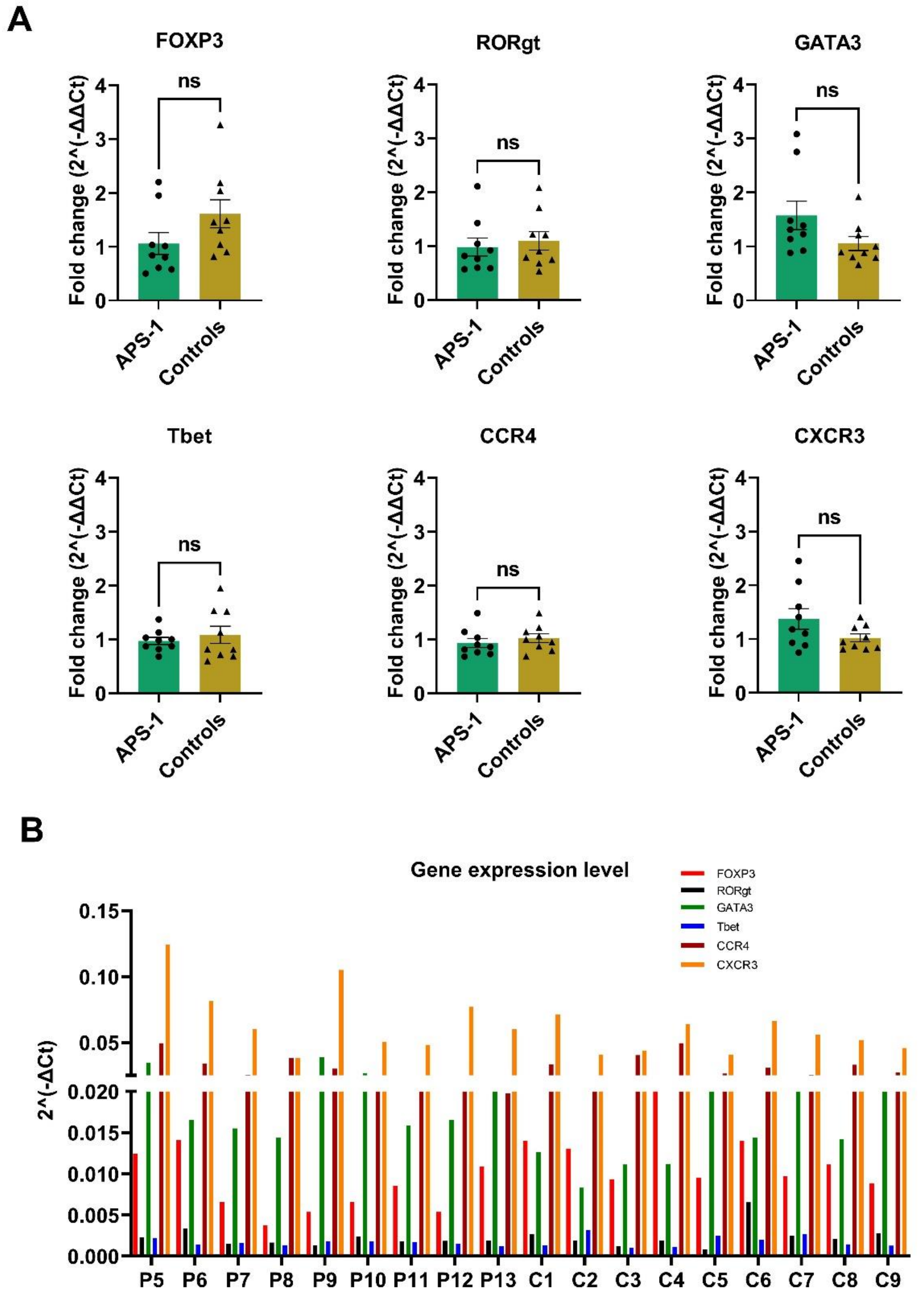

SYBR Green QPCRs were run on expanded Tregs from 9 APS-1 patients and 9 healthy controls. A. Fold change values for patients vs. healthy controls. The boxes show the mean, and the standard deviation is represented. B. Gene expression level of each gene in all samples.

Figure S4. Expanded Treg immune panel single-cell profiles of APS-1 patients (N=8) and healthy controls (N=8). ***Related to main Figure 2.***

A

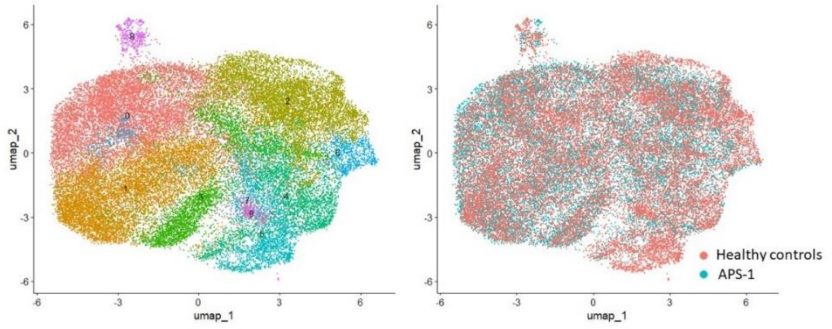

B

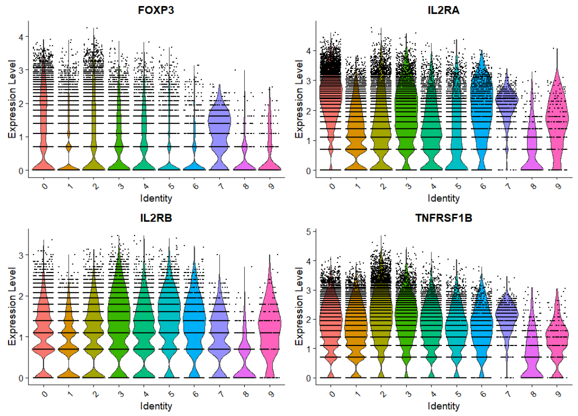

C

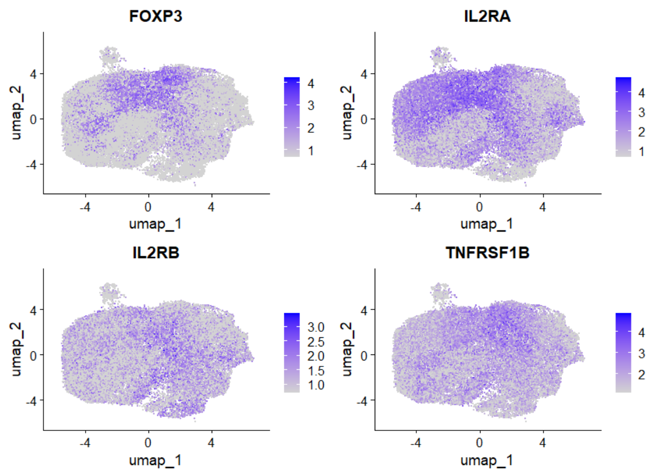

(A) UMAP projection of the immune panel based on expanded Tregs from 8 APS-1 patients and 8 healthy controls identified ten different clusters of Tregs when profiles were overlaid (Left: The clusters in both groups. Right: Distribution of cells in the UMAP shown for patients (green) versus controls (red)). (B) Violin plots on expression of the four Treg markers FOXP3, IL2RA, IL2RB and TNFRSF1B in the 10 clusters. C. Feature plots of the Treg characteristic markers FOXP3, IL2RA, IL2RB and TNFRSF1B in the 10 clusters. The figure shows the 10% of cells that express the highest levels of the markers in purple.

Figure S5. Heat map of the top 15 most differentially expressed genes in expanded Tregs between APS-1 patients (N=8) and healthy controls (N=8). ***Related to main Figure 2.***

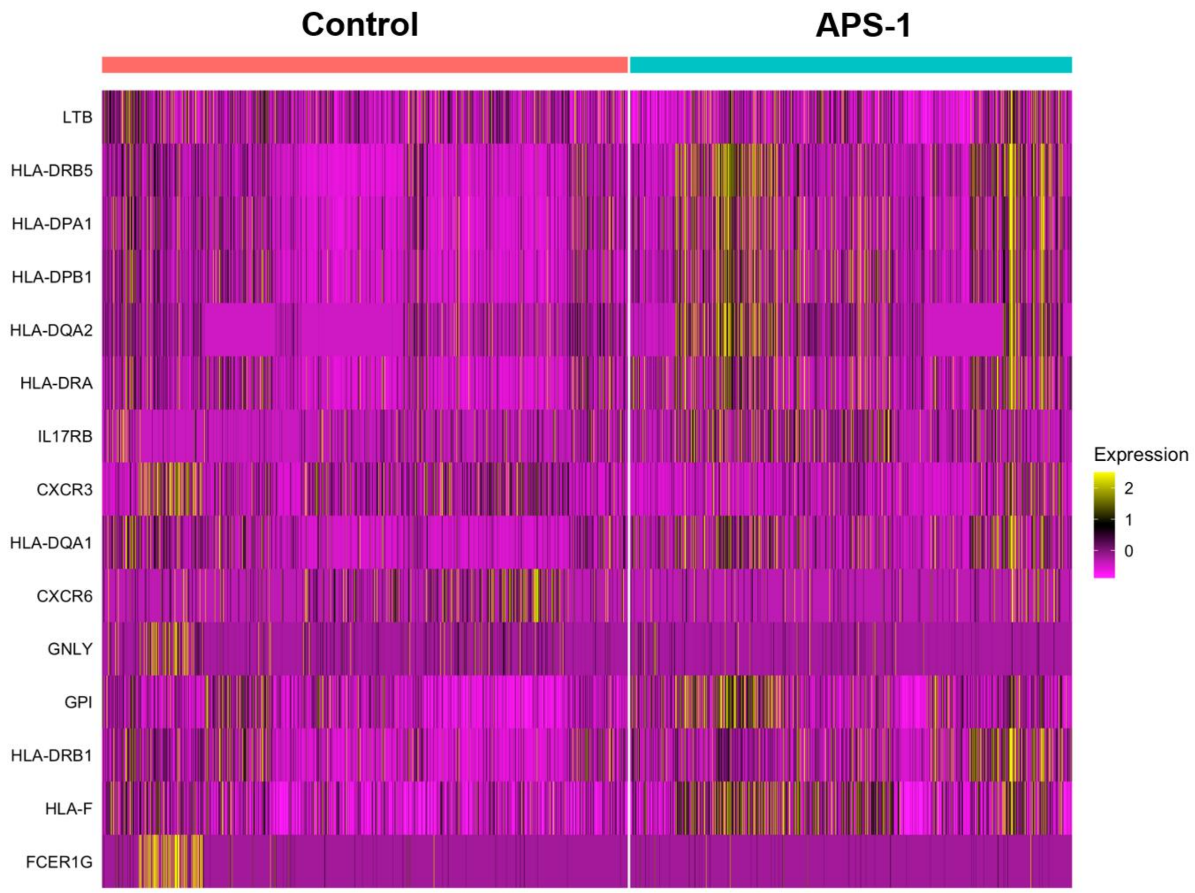

Heat map for each dataset healthy controls and APS-1 patients depicting the gene expression level per cell for each of the top 15 most expressed genes. It was noticed that for each gene, the expression might change significantly from one cell to another.

Figure S6. Ingenuity pathway analysis of significant differentiated genes from expanded Tregs from APS-1 patients (N=8) and healthy controls (N=8). ***Related to main Figure 2.***

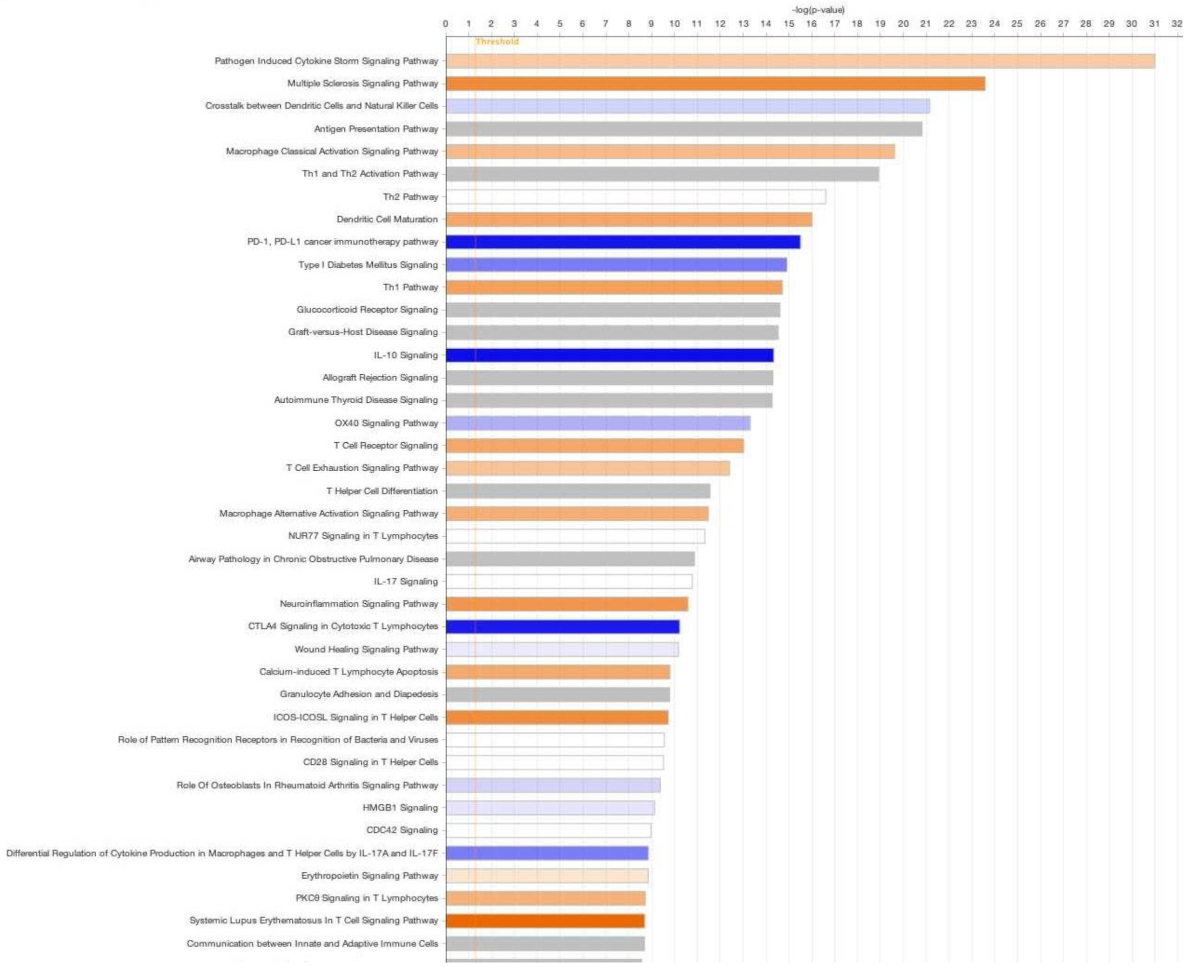

Inguienity pathway analysis of significant differentially expressed genes in expanded Tregs between APS-1 patients and healthy controls.

Figure S7. **Differentially expressed genes in both freshly sorted and expanded Tregs.** ***Related to main Figure 1 and 2.***

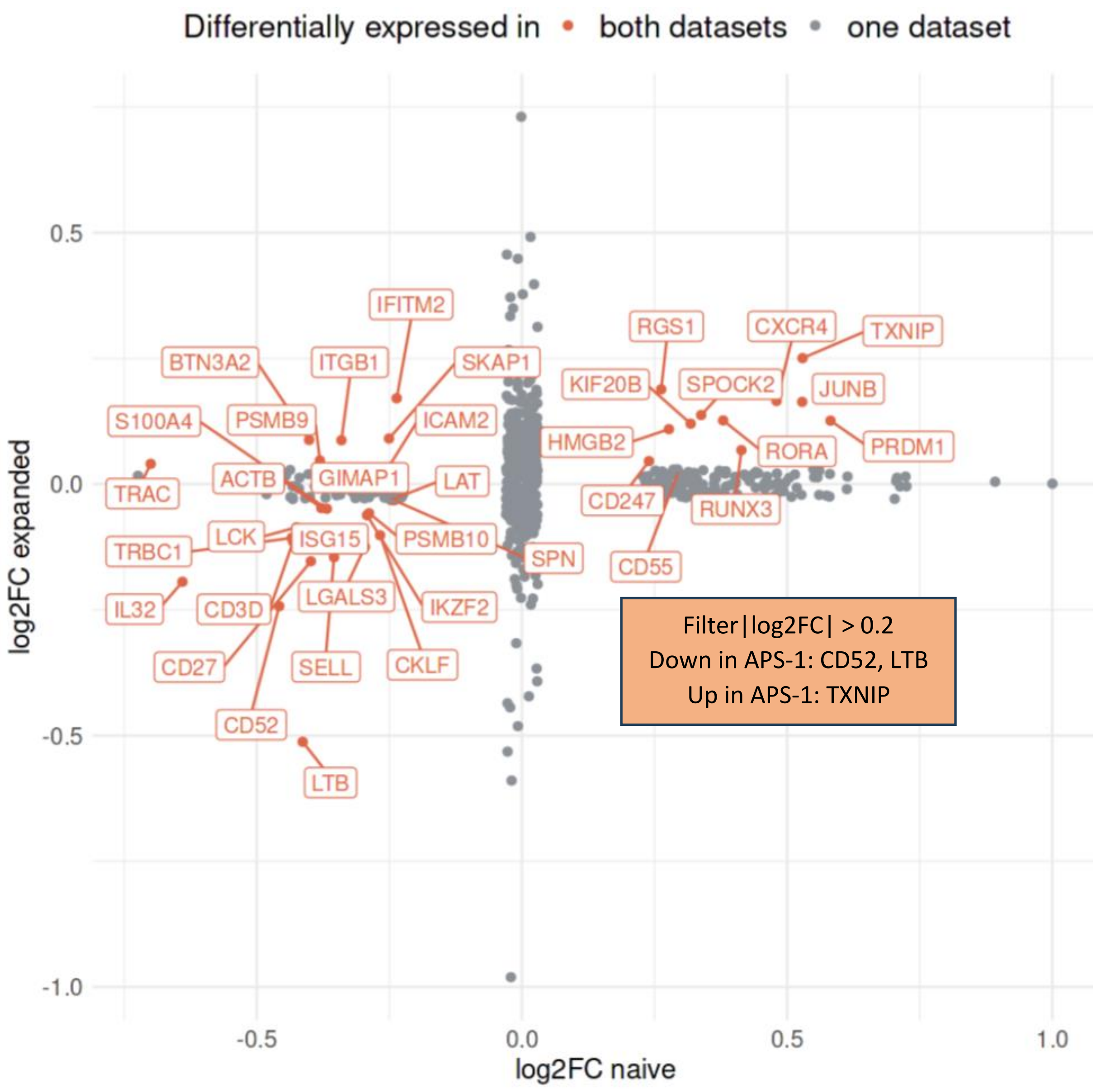

All differentially expressed genes present in both the freshly sorted Tregs (x-axis) and in the expanded Tregs immune panel cohort (y-axis) are highlighted in red. Grey circles are genes that only show differential expressions between APS-1 patients and controls in one of the datasets. If applying a filter of |log2FC| > 0.2, then only three genes are shared between our results: *CD52* (down in patients), *TXNIP* (up in patients) *and LTB* (down in patients).

Figure S8. TCR clonotype abundance, count and gene usage for APS-1 patients (N=7) and healthy controls (N=9) for expanded Tregs. ***Related to main Figure 3.***

A.

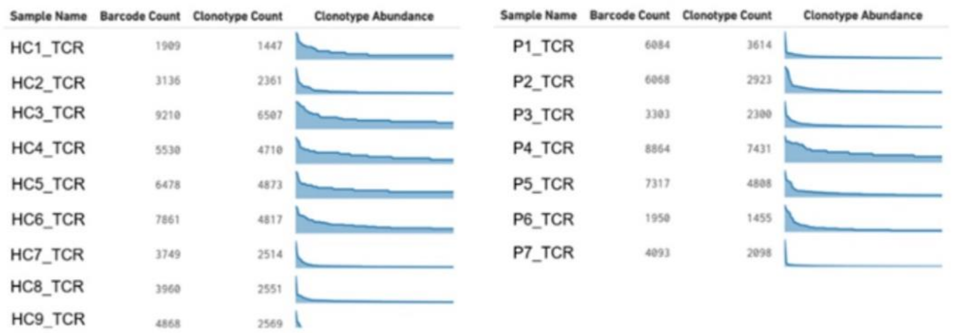

Sample Name	Barcode Count	Clonotype Count	Clonotype Abundance
HC1_TCR	1909	1447	
HC2_TCR	3136	2361	
HC3_TCR	9210	6507	
HC4_TCR	5530	4710	
HC5_TCR	6478	4873	
HC6_TCR	7861	4817	
HC7_TCR	3749	2514	
HC8_TCR	3960	2551	
HC9_TCR	4868	2569	

Sample Name	Barcode Count	Clonotype Count	Clonotype Abundance
P1_TCR	6084	3614	
P2_TCR	6068	2923	
P3_TCR	3303	2300	
P4_TCR	8864	7431	
P5_TCR	7317	4808	
P6_TCR	1950	1455	
P7_TCR	4093	2098	

B.

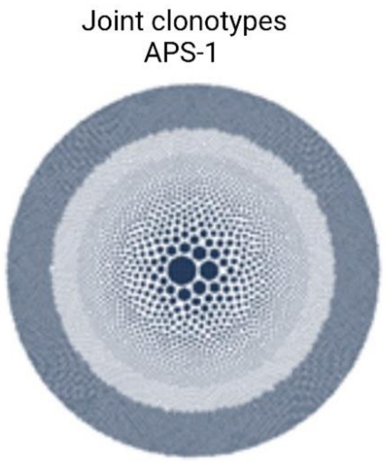

C.

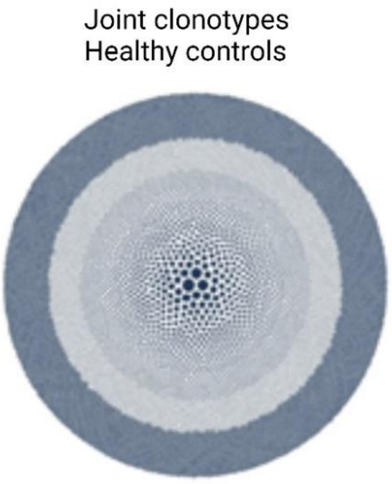

(A) Clonotype abundance and count per sample.
(B) Representation of joint clonotypes for APS-1 patients.
(C) Representation of joint clonotypes for healthy controls.

Figures taken from Loupe VSJ Browsers, 10x Genomics. Figure created by Biorender.com.

Figure S9. V, D and J gene usage per sample for expanded Tregs from APS-1 patients (N=7). ***Related to main Figure 3.***

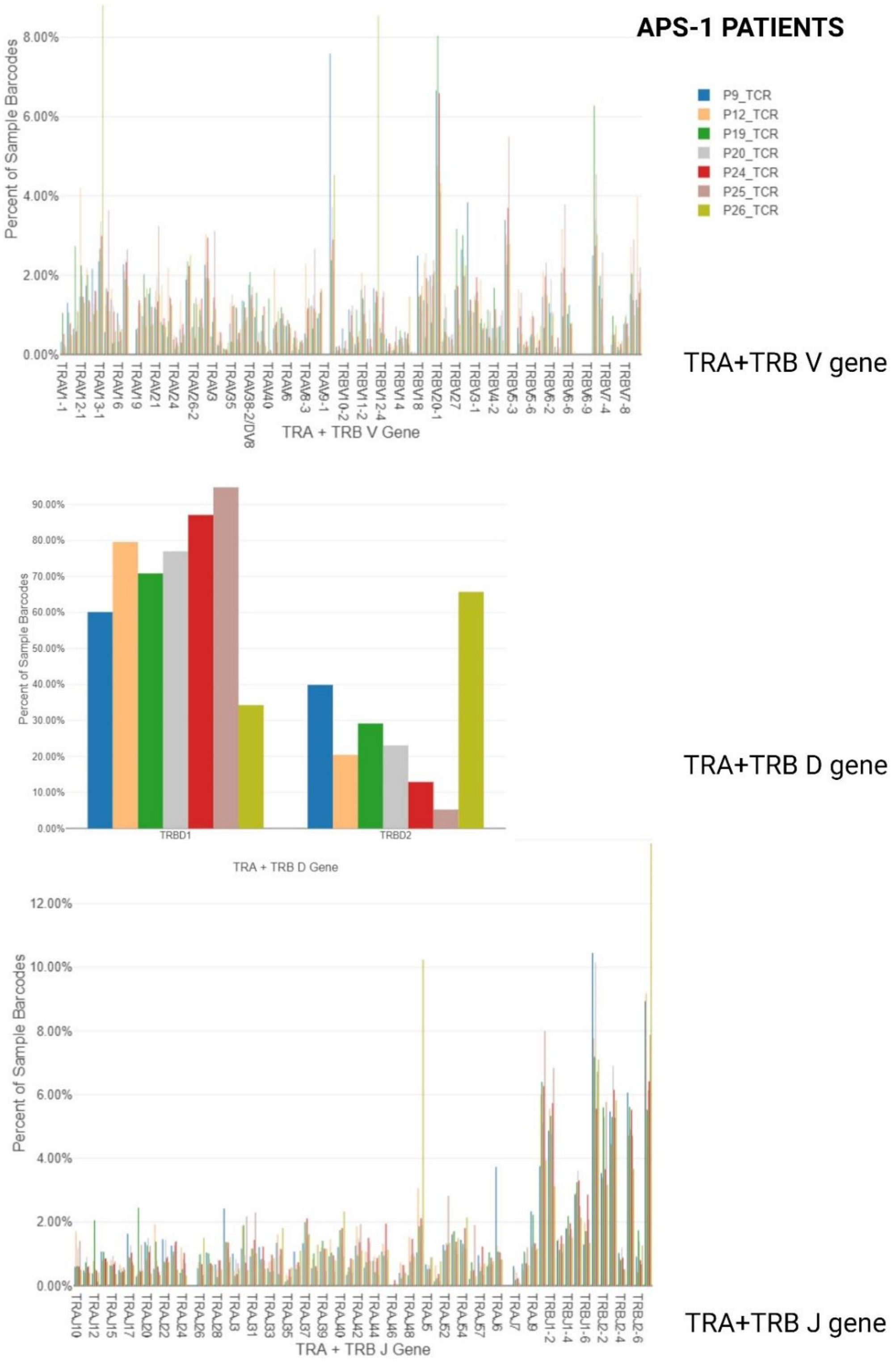

V, D and J gene usage per sample for APS-I patients. Two patient samples did not show a high enough diversity, which was a prerequisite for analysis, and was removed from the dataset. Figures taken from Loupe VSJ Browsers, 10x Genomics. Figure created by Biorender.com.

Figure S10. V, D and J gene usage per sample for expanded Tregs from healthy controls (N=9). ***Related to main Figure 3.***

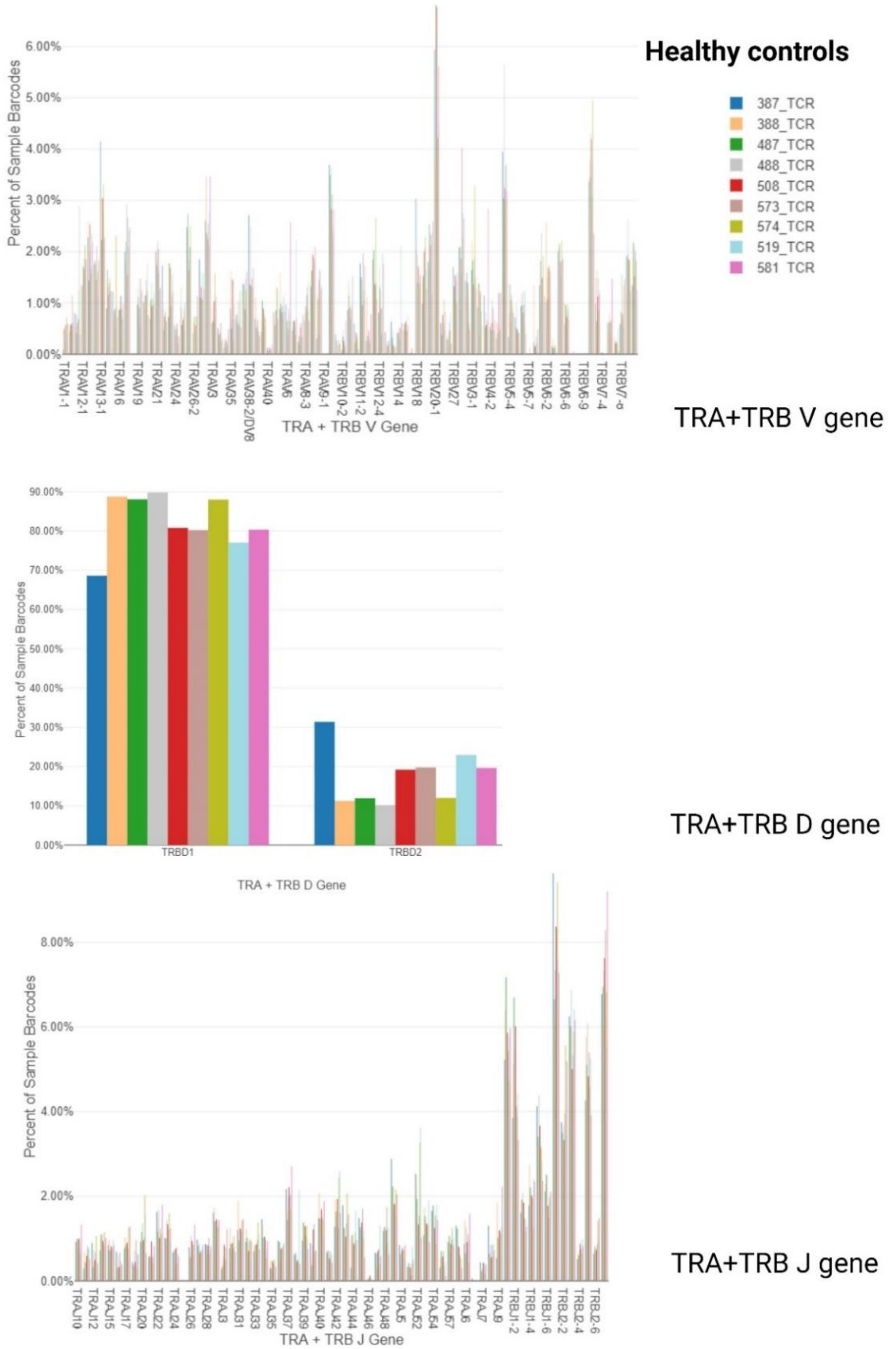

V, D and J gene usage per sample for APS-I patients. Figures taken from Loupe VSJ Browsers, 10x Genomics. Figure created by Biorender.com.

Figure S11. Individual TCR clonotypes within expanded Tregs for healthy controls (N=8, C1-C8). ***Related to main Figure 3.***

c1

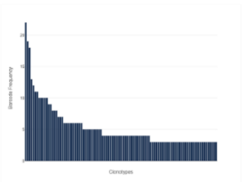

	V	D	J	CDR3	#
TRB	TRBV18	TRBD2	TRBJ2-1	CASSPQGLAGVGEQFF	22
TRB	TRBV12-3		TRBJ1-3	CASLMVGGNTIYF	19
TRA	TRAV26-1		TRAJ22	CIVSGSARQLTF	
TRB	TRBV13		TRBJ2-7	CASSLTILPEQYF	18
TRA	TRAV25		TRAJ37	CAGSSSNTGKLIF	
TRB	TRBV18	TRBD2	TRBJ2-1	CASSPQGLAGVGEQFF	13
TRA	TRAV29/D...		TRAJ36	CAASYQTGANNLFF	
TRB	TRBV18		TRBJ2-5	CASSPPGTVKETQYF	12
TRA	TRAV14/D...		TRAJ49	CAMREGIPNTGNQFYF	

c2

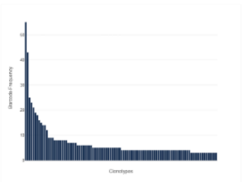

	V	D	J	CDR3	#
TRB	TRBV5-8		TRBJ2-5	CASSRSGGTQETQYF	55
TRA	TRAV23/D...		TRAJ52	CAANSHAGGTSYGKL...	
TRB	TRBV3-1		TRBJ1-4	CASSPYRTRGEKLFF	43
TRA	TRAV29/D...		TRAJ42	CAASGSGGSQGNLIF	
TRB	TRBV9		TRBJ2-2	CASSDEGQASTGELFF	25
TRA	TRAV5		TRAJ6	CAEKSGGSYIPTF	
TRB	TRBV12-4	TRBD1	TRBJ2-3	CASIPGPGTGGGSTD...	23
TRA	TRAV12-3		TRAJ9	CAMRRTGGFKTIF	
TRB	TRBV6-2		TRBJ2-3	CASSYSQPTQYF	21
TRA	TRAV27		TRAJ29	CAGAYSGNTPLVF	

c3

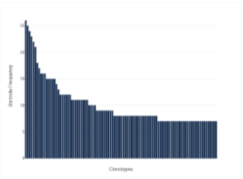

	V	D	J	CDR3	#
TRB	TRBV12-4		TRBJ2-7	CASTAGPGAGMYEQYF	
TRB	TRBV20-1	TRBD1	TRBJ2-3	CSASGPGGLGTDTQYF	26
TRA	TRAV23/D...		TRAJ42	CAATVLGSQGNLIF	
TRB	TRBV5-4		TRBJ2-5	CASSSRSGGQETQYF	25
TRA	TRAV8-1		TRAJ38	CAVNAGAGNNRKLIW	
TRB	TRBV20-1		TRBJ2-1	CSATVDRRTYNEQFF	24
TRA	TRAV12-1		TRAJ10	CVVATGGGNKLTF	
TRB	TRBV12-3	TRBD1	TRBJ2-1	CASSAGTAGFF	23
TRA	TRAV20		TRAJ13	CAVSGSGGYQKVTF	
TRB	TRBV5-1		TRBJ2-7	CATTPGPTSYEQYF	22
TRA	TRAV1-1		TRAJ22	CAVRAVSSGSARQLTF	

c4

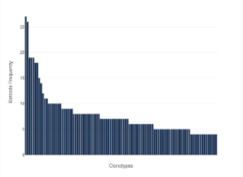

	V	D	J	CDR3	#
TRB	TRBV9		TRBJ2-1	CASSARSYEQFF	27
TRA	TRAV17		TRAJ18	CATVPADRGSTLGRL...	
TRB	TRBV20-1		TRBJ2-5	CSAHRAGGVQETQYF	26
TRA	TRAV17		TRAJ26	CAPQAYGQNFVF	
TRB	TRBV19		TRBJ1-5	CASWDISEGPQHF	19
TRA	TRAV20		TRAJ42	CAIMNYGGSQGNLIF	
TRB	TRBV7-2		TRBJ2-5	CASSARGGETQYF	
TRB	TRBV5-1		TRBJ1-1	CASSLGADFNTEAFF	19
TRA	TRAV27		TRAJ32	CAGESWGATNKLIF	
TRA	TRAV12-3		TRAJ37	CAMMSSSNTGKLIF	19
TRB	TRBV20-1	TRBD1	TRBJ1-5	CSARTGGSGNQPQHF	18
TRA	TRAV8-4		TRAJ15	CAVSNQAGTALIF	

c5

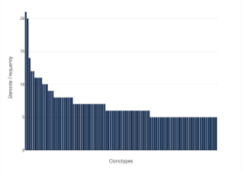

	V	D	J	CDR3	#
TRB	TRBV12-4		TRBJ1-5	CASSFRPGGQPQHF	21
TRA	TRAV23/D...		TRAJ23	CAASSRGNQGGKLIF	
TRB	TRBV20-1		TRBJ2-1	CSARDLTSGTRSSYN...	20
TRA	TRAV13-1		TRAJ43	CAAGLNNNNDMRF	
TRB	TRBV7-2		TRBJ2-4	CASSPRSRDIQYF	14
TRA	TRAV20		TRAJ7	CAVQNGNNRLAF	
TRB	TRBV4-1		TRBJ1-2	CASSPRLQGYGYTF	12
TRA	TRAV27		TRAJ29	CAGAPPGNTPLVF	
TRB	TRBV20-1		TRBJ1-1	CSARLGQPNTEAFF	12
TRA	TRAV8-2		TRAJ5	CVVSDRDTGRRALTF	

c6

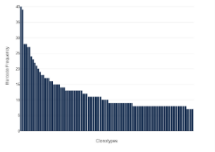

	V	D	J	CDR3	#
TRB	TRBV6-5		TRBJ1-1	CAEGQFMNTEAFF	
TRB	TRBV7-2		TRBJ1-4	CASSLSGREKLFF	40
TRA	TRAV17		TRAJ22	CATGPISSGSARQLTF	
TRB	TRBV7-3		TRBJ2-7	CASSVQPGAGEQYF	39
TRA	TRAV13-1		TRAJ48	CAASKEGLGNEKLTF	
TRB	TRBV28		TRBJ1-2	CASSSQGAGYTF	
TRA	TRAV8-3		TRAJ43	CAVATLYNNNDMRF	28
TRA	TRAV8-3		TRAJ40	CAVGVHSGTYKYIF	
TRB	TRBV19		TRBJ2-2	CASKSDPVWELFF	28
TRA	TRAV1-2		TRAJ39	CAVRDQDAGNMLTF	
TRB	TRBV9		TRBJ2-5	CASSRLLEETQYF	27
TRA	TRAV2		TRAJ37	CAVVAGNTGKLIF	

c7

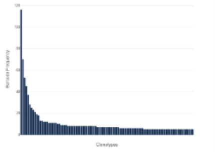

	V	D	J	CDR3	#
TRB	TRBV3-1		TRBJ2-1	CASSHSGGIHEQFF	
TRA	TRAV14/D...		TRAJ42	CAFPGGSQGNLIF	116
TRA	TRAV14/D...		TRAJ52	CAMREFYAGGTSYGK...	
TRB	TRBV5-1		TRBJ2-2	CASSPEAGELFF	70
TRA	TRAV13-2		TRAJ43	CAERNNDMRF	
TRB	TRBV12-3		TRBJ2-1	CASSTGTAEQFF	53
TRA	TRAV12-1		TRAJ49	CVVCRNTGNQFYF	
TRA	TRAV10		TRAJ20	CVVSANSNDYKLSF	45
TRB	TRBV7-2	TRBD1	TRBJ1-6	CASSQGQGGNSPLHF	37
TRA	TRAV41		TRAJ57	CAVQVGKLVF	
TRB	TRBV7-2		TRBJ2-1	CASSPEGGSYNEQFF	28
TRA	TRAV1-1		TRAJ26	CAVRDHYGQNFVF	

c8

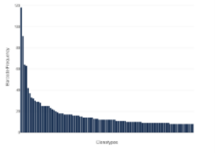

	V	D	J	CDR3	#
TRB	TRBV5-1	TRBD1	TRBJ2-7	CASSSGQGKLYEQYF	118
TRA	TRAV6		TRAJ37	CALGKGSNTGKLIF	
TRB	TRBV4-1		TRBJ1-6	CASSQAYKGAFESSP...	91
TRA	TRAV6		TRAJ9	CALAYTGGFKTIF	
TRB	TRBV4-1		TRBJ2-1	CASSQDYRGKINEQFF	64
TRA	TRAV12-2		TRAJ22	CAVNGASGSARQLTF	
TRB	TRBV6-5		TRBJ1-1	CASSYEGISINTEAFF	63
TRA	TRAV25		TRAJ26	CAGGNYGQNFVF	
TRB	TRBV2		TRBJ2-2	CANQRERAGELFF	42
TRA	TRAV29/D...		TRAJ42	CAASLNYGGSQGNLIF	

The table for each individual shows the 5 most used clonotypes and the histogram visualises the abundancy of the 100 most used clonotypes.

Figure S12. Individual TCR clonotypes within expanded Tregs for APS-1 patients (N=8, P1-P8). ***Related to main Figure 3.***

p1

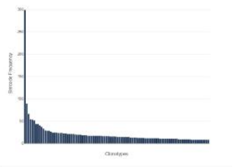

	V	D	J	CDR3	#
TRB	TRBV29-1	TRBD2	TRBJ2-1	CSATTGTSGNNEQFF	299
TRA	TRAV9-2		TRAJ6	CALWGGSYIPTF	
TRB	TRBV7-3		TRBJ2-1	CASSTRTSGGNEQFF	89
TRA	TRAV12-3		TRAJ40	CAMSLSGTYKYIF	
TRA	TRAV9-2		TRAJ9	CALSTGGFKTIF	66
TRB	TRBV20-1		TRBJ2-3	CSARPAGGGFTDTQYF	54
TRA	TRAV1-2		TRAJ17	CAVRTPSGNKLTF	
TRB	TRBV19		TRBJ2-7	CASSAFGRGSSYEQYF	53
TRA	TRAV9-2		TRAJ9	CALSTGGFKTIF	

p2

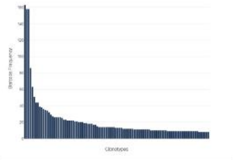

	V	D	J	CDR3	#
TRB	TRBV9		TRBJ1-2	CASSVDPEGYGYTF	163
TRA	TRAV8-3		TRAJ10	CAVGGRGGGNKLTF	
TRA	TRAV12-1		TRAJ30	CVGNRDDKIIF	
TRB	TRBV7-9	TRBD1	TRBJ2-7	CASTGDIYEQYF	158
TRA	TRAV12-1		TRAJ21	CVVNRDFNKFYF	
TRB	TRBV19		TRBJ2-7	CASSTLDSLSYEQYF	158
TRA	TRAV41		TRAJ49	CAVLARTGNQFYF	
TRB	TRBV27		TRBJ2-2	CASSPSYGGTGELFF	86
TRA	TRAV22		TRAJ4	CAVERLGGYNKLIF	
TRB	TRBV11-2		TRBJ2-3	CASSPKNPTDTQYF	63
TRA	TRAV23/D...		TRAJ48	CAASTNFGNEKLTF	
TRA	TRAV22		TRAJ24	CAVITDSWGKLQF	

p3

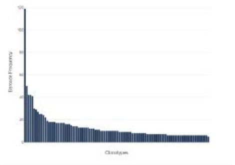

	V	D	J	CDR3	#
TRB	TRBV27	TRBD2	TRBJ2-2	CASSTGLAGLRNTGE...	119
TRA	TRAV10		TRAJ18	CVVSGGGSTLGRLYF	
TRB	TRBV4-2		TRBJ2-1	CASSPVGPVREQFF	50
TRA	TRAV39		TRAJ30	CAVESMNRDDKIIF	
TRB	TRBV20-1		TRBJ1-4	CSARGRQNNEKLFF	42
TRA	TRAV12-1		TRAJ21	CVVNMNNFNKFYF	
TRB	TRBV11-1		TRBJ1-1	CASSRHMNTEAFF	42
TRA	TRAV26-1		TRAJ26	CIVRVLDGQNFVF	
TRA	TRAV40		TRAJ12	CLLGDSLAMDSSYKL...	
TRB	TRBV20-1		TRBJ2-5	CSWDNRETQYF	41
TRA	TRAV38-2...		TRAJ30	CAYRVNRDDKIIF	

p4

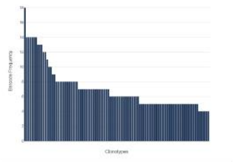

	V	D	J	CDR3	#
TRB	TRBV20-1	TRBD1	TRBJ2-7	CSARISGTGTYEQYF	18
TRA	TRAV29/D...		TRAJ31	CAASAAWARLMF	
TRB	TRBV20-1		TRBJ2-1	CSARAGATVYNEQFF	14
TRA	TRAV12-2		TRAJ24	CAVRRTTDSWGKLQF	
TRB	TRBV24-1		TRBJ2-2	CATRGGPNTGELFF	14
TRA	TRAV13-2		TRAJ23	CAENTELIF	
TRB	TRBV7-3		TRBJ2-1	CASSQAGGLYNEQFF	14
TRA	TRAV2		TRAJ39	CAVPNAGNMLTF	
TRA	TRAV20		TRAJ3	CAVPWYSSASKIIF	
TRB	TRBV7-2		TRBJ1-6	CASSLEGQLNSPLHF	14
TRA	TRAV1-2		TRAJ22	CAVRALSGSARQLTF	

p5

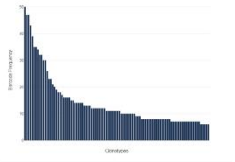

	V	D	J	CDR3	#
TRB	TRBV5-4		TRBJ1-6	CASSLGALINSPLHF	50
TRA	TRAV13-2		TRAJ20	CAELNDYKLSF	
TRB	TRBV20-1		TRBJ2-1	CSARPAVSYNEQFF	47
TRA	TRAV17		TRAJ58	CATDGETSGSRLTF	
TRB	TRBV2		TRBJ2-2	CASRRGTANTGELFF	47
TRA	TRAV38-2...		TRAJ53	CAYRRSGGSNYKLTF	
TRB	TRBV12-3		TRBJ2-7	CASSPNSGGASYEQYF	43
TRA	TRAV26-2		TRAJ40	CILKSGSGTYKYIF	
TRB	TRBV20-1		TRBJ1-2	CSARDGRGHGYTF	39
TRA	TRAV6		TRAJ36	CAPLSGANNLFF	

p6

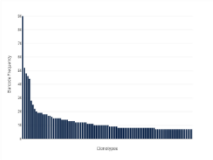

	V	D	J	CDR3	#
TRB	TRBV20-1	TRBD1	TRBJ1-2	CSARVGQGARGYTF	90
TRA	TRAV14/D...		TRAJ37	CAMRYGSSNTGKLIF	
TRB	TRBV29-1		TRBJ2-2	CSSRGAANTGELFF	52
TRA	TRAV3		TRAJ9	CAVRDPNTGGFKTIF	
TRB	TRBV7-2		TRBJ2-5	CASSLGMAGGSETQYF	48
TRA	TRAV29/D...		TRAJ41	CAASLPGYALNF	
TRA	TRAV2		TRAJ45	CAVEDPAGGGADGLTF	
TRB	TRBV7-2		TRBJ2-3	CAITTRADTQYF	46
TRA	TRAV5		TRAJ45	CAEKGRGSGGGADGL...	
TRA	TRAV9-2		TRAJ24	CALRSDSWGKLQF	
TRB	TRBV27		TRBJ2-3	CASSSRPRLAGQILS...	44
TRA	TRAV38-2...		TRAJ57	CAYRTRDVGSEKLVF	

p7

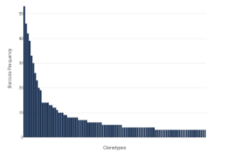

	V	D	J	CDR3	#
TRB	TRBV5-1		TRBJ2-2	CASSLEPGTYTGELFF	53
TRA	TRAV3		TRAJ31	CAVRATSTGFLGLMF	
TRB	TRBV7-3	TRBD1	TRBJ2-2	CASSRDRVGGELFF	46
TRA	TRAV13-2		TRAJ56	CAEMTGANSKLTF	
TRB	TRBV7-9	TRBD1	TRBJ2-1	CASSPTLGAGENNEQ...	42
TRA	TRAV8-4		TRAJ52	CAVISGGTSYGKLTF	
TRB	TRBV2	TRBD1	TRBJ2-7	CASSVSGQGAYEQYF	39
TRA	TRAV13-2		TRAJ30	CAESLFLNRDDKIIF	
TRB	TRBV7-2		TRBJ1-2	CASSLDRVQGNYGYTF	33

p8

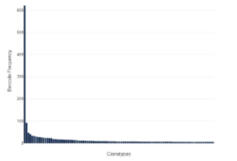

	V	D	J	CDR3	#
TRB	TRBV12-3	TRBD2	TRBJ2-7	CASSPNPGLAGEQYF	621
TRA	TRAV13-1		TRAJ49	CAASSRNQFYF	
TRB	TRBV15		TRBJ2-3	CATSRVRESDTQYF	92
TRA	TRAV9-2		TRAJ26	CALSVLPRDNYGQNF...	
TRB	TRBV18	TRBD2	TRBJ2-5	CASSPRASGSGETQYF	46
TRA	TRAV13-1		TRAJ40	CAASTTSGTYKYIF	
TRB	TRBV5-8		TRBJ1-1	CASSLGLLNTEAFF	40
TRA	TRAV9-2		TRAJ34	CALPGSYNTDKLIF	
TRB	TRBV4-2		TRBJ1-3	CASSQAPDPGNTIYF	33
TRA	TRAV41		TRAJ49	CAVRSFPPGNQFYF	

The table for each individual shows the 5 most used clonotypes and the histogram visualises the abundancy of the 100 most used clonotypes.

Figure S13. The gating strategy for flow cytometry characterization of expanded Tregs. ***Related to main Figure 4.***

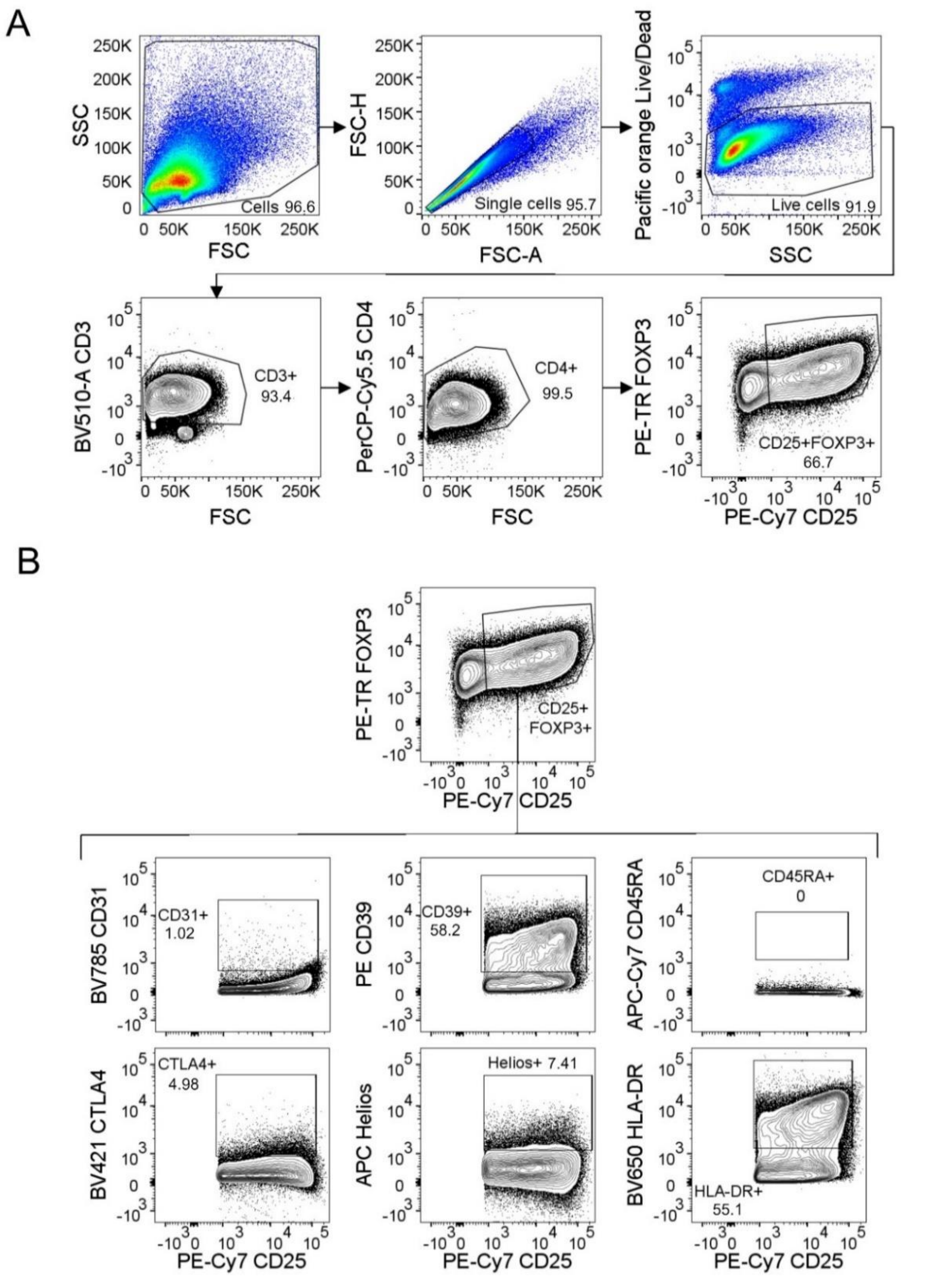

The gating strategy is shown for a representative APS-1 patient. (A) Samples were gated as single cells, live cells, and CD3 positive cells, and further as CD4 positive cells. The Treg fraction was gated as CD4+CD25+FOXP3+ cells. (B) Within the CD4+CD25+FOXP3+ Treg fraction, samples were gated for the functional markers CD31, CD39, CD45RA, CTLA, Helios and HLA-DR.

Figure S14. Flow cytometry expression of (A) CD45RA, (B) CTLA4, (C) CD39 and (D) HLA-DR within expanded Tregs. ***Related to main Figure 4.***

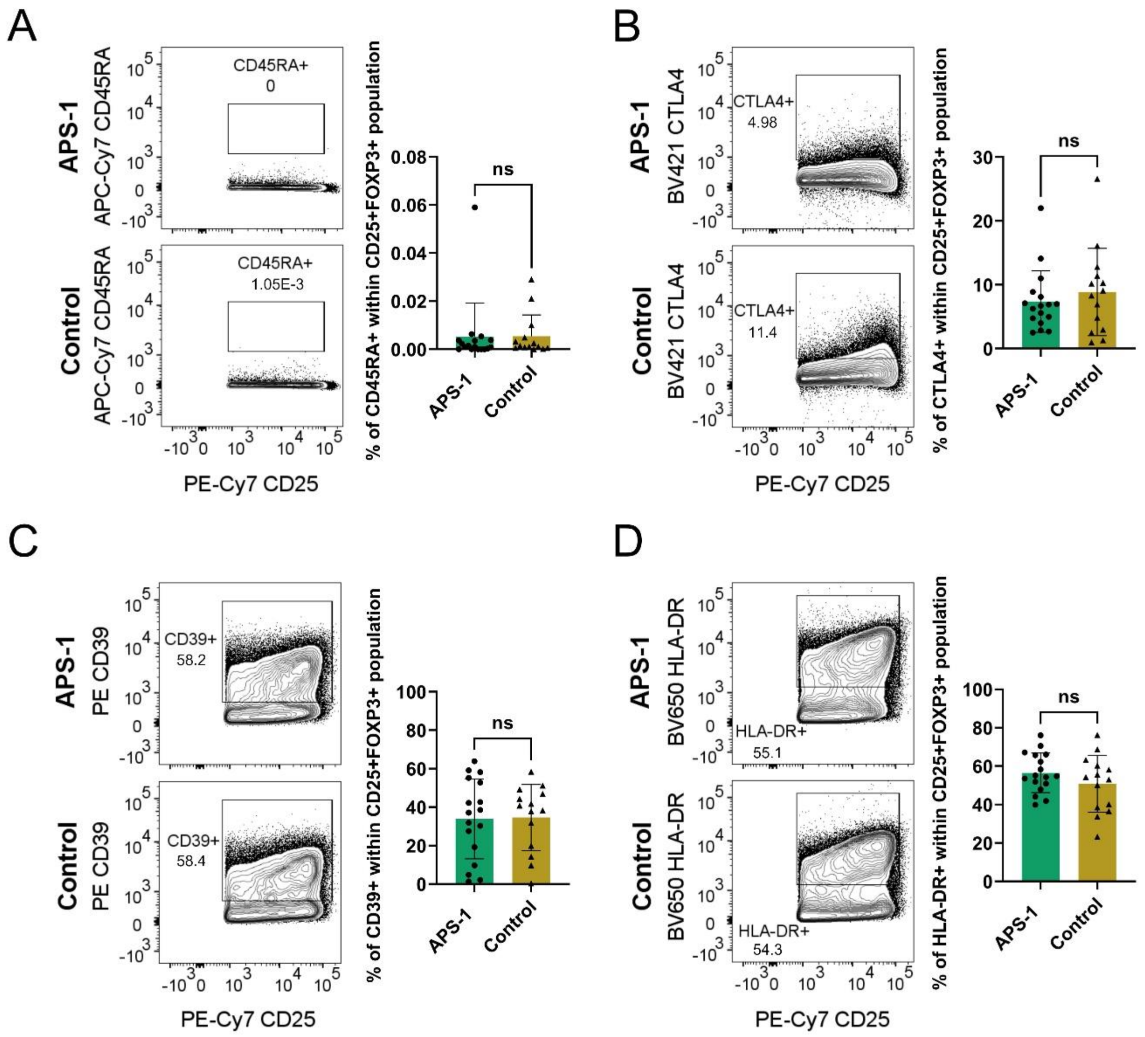

Flow figures are shown for a representative APS-1 patient and a healthy control for each marker. *represents a p-value < 0.05, calculated using an unpaired, parametric t-test. ns; non-significant.

Figure S15. CyTOF batch correction (for expanded Tregs). ***Related to main Figure 5.***

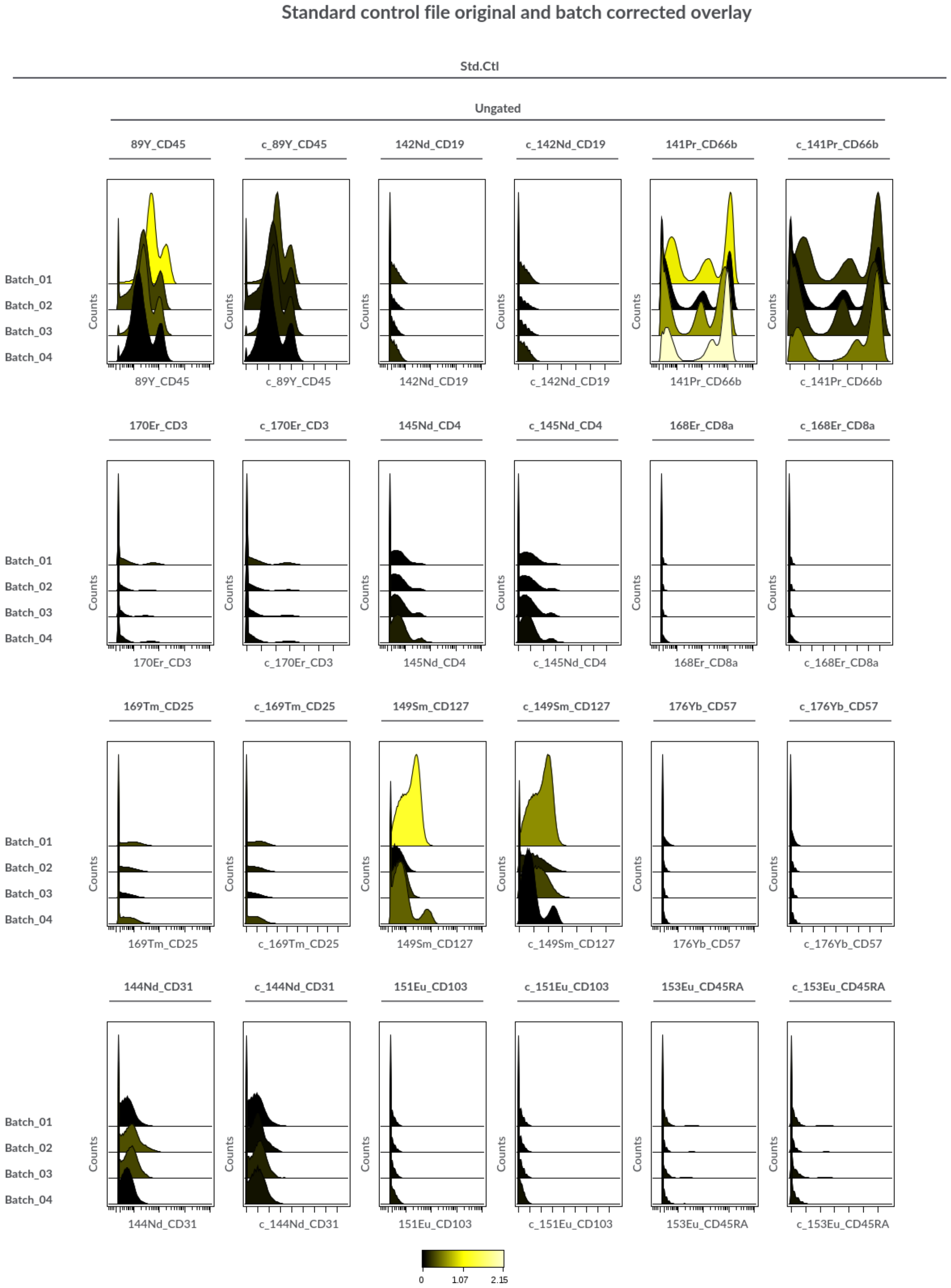

12 CyTOF channels before and after (c_) batch correction based on an internal control included in all four batches.

Figure S16. The gating strategy for CyTOF characterisation of expanded Tregs. ***Related to main Figure 5.***

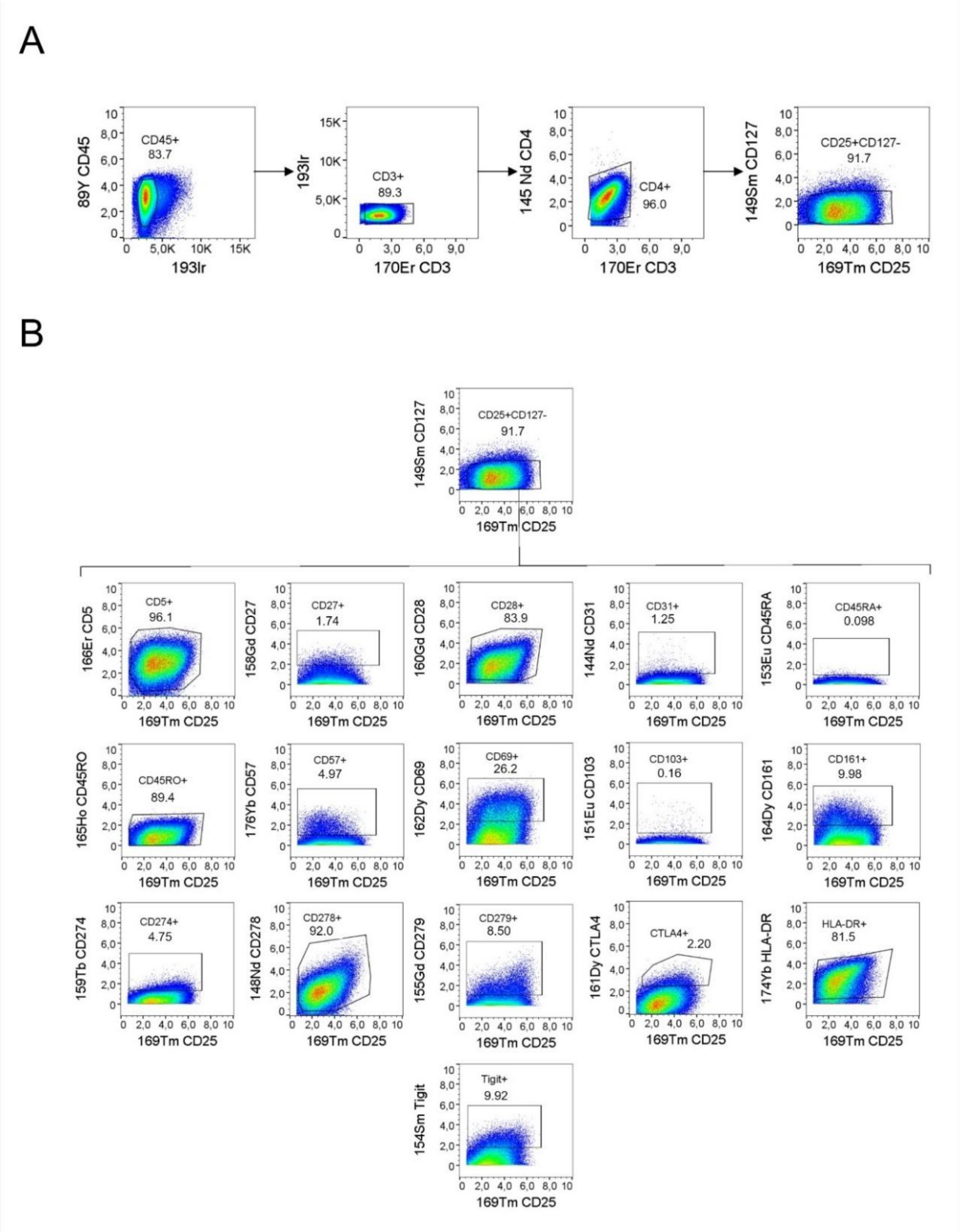

Gating strategy and data clean up shown for a representative APS-1 patient. (A) After batch correction, samples were gated as CD45 positive cells, CD3 positive cells, and further as CD4 positive cells. The Treg fraction was gated as CD4+CD25+CD127- cells. (B) Within the CD4+CD25+CD127- Treg fraction, samples were gated for the markers CD5, CD27, CD28, CD31, CD45RA, CD45RO, CD57, CD69, CD103, CD161, CD274, CD278, CD279, CTLA4, HLA-DR and Tigit.

Figure S17. Heat map showing the expression of all CyTOF panel markers for expanded Tregs (N=17 APS-1 patients and 17 healthy controls). ***Related to main Figure 5.***

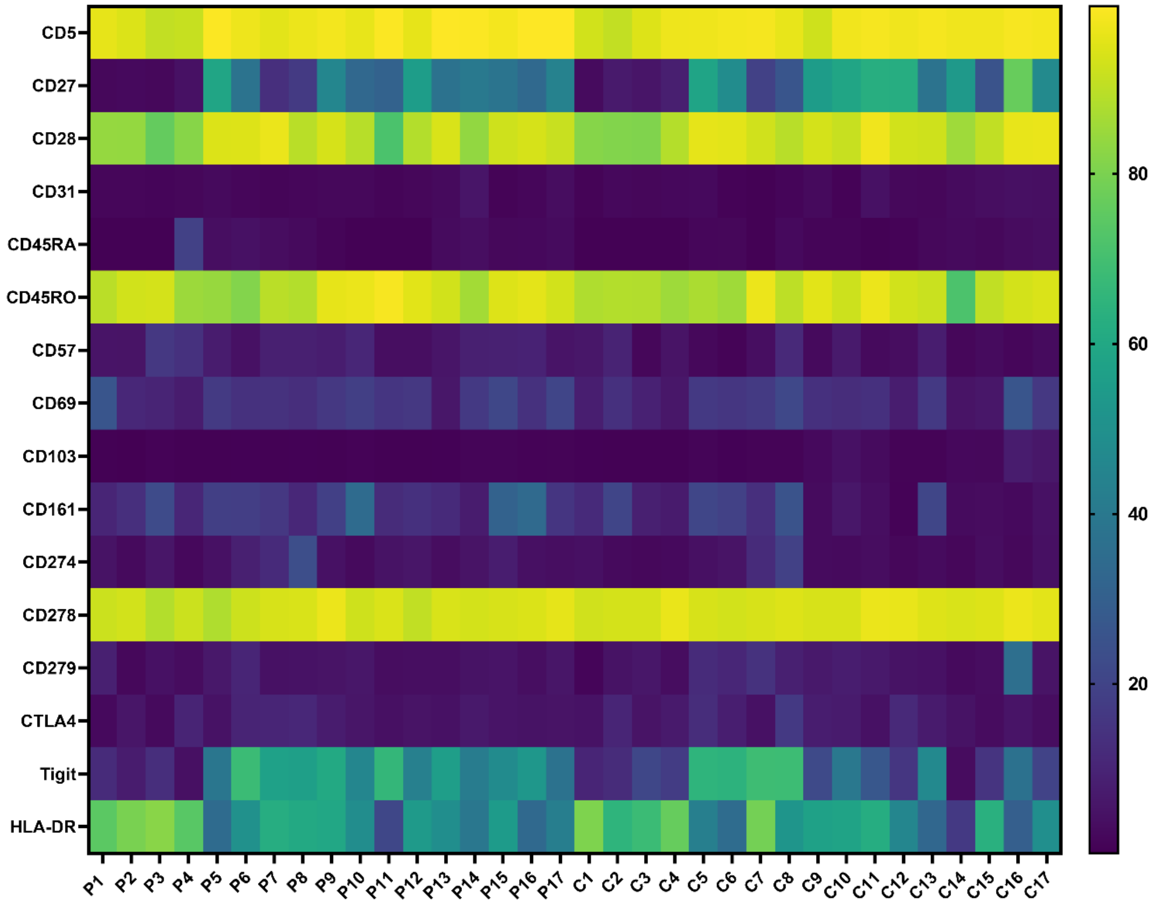

The expression of all markers within the CD4+CD25+ CD127^{low}population for all APS-1 patients and all healthy controls is included. P; patient, C; control. The scale is based on frequencies and is shown to the right.

Figure S18. Treg suppression assay gating strategy. ***Related to main Figure 6.***

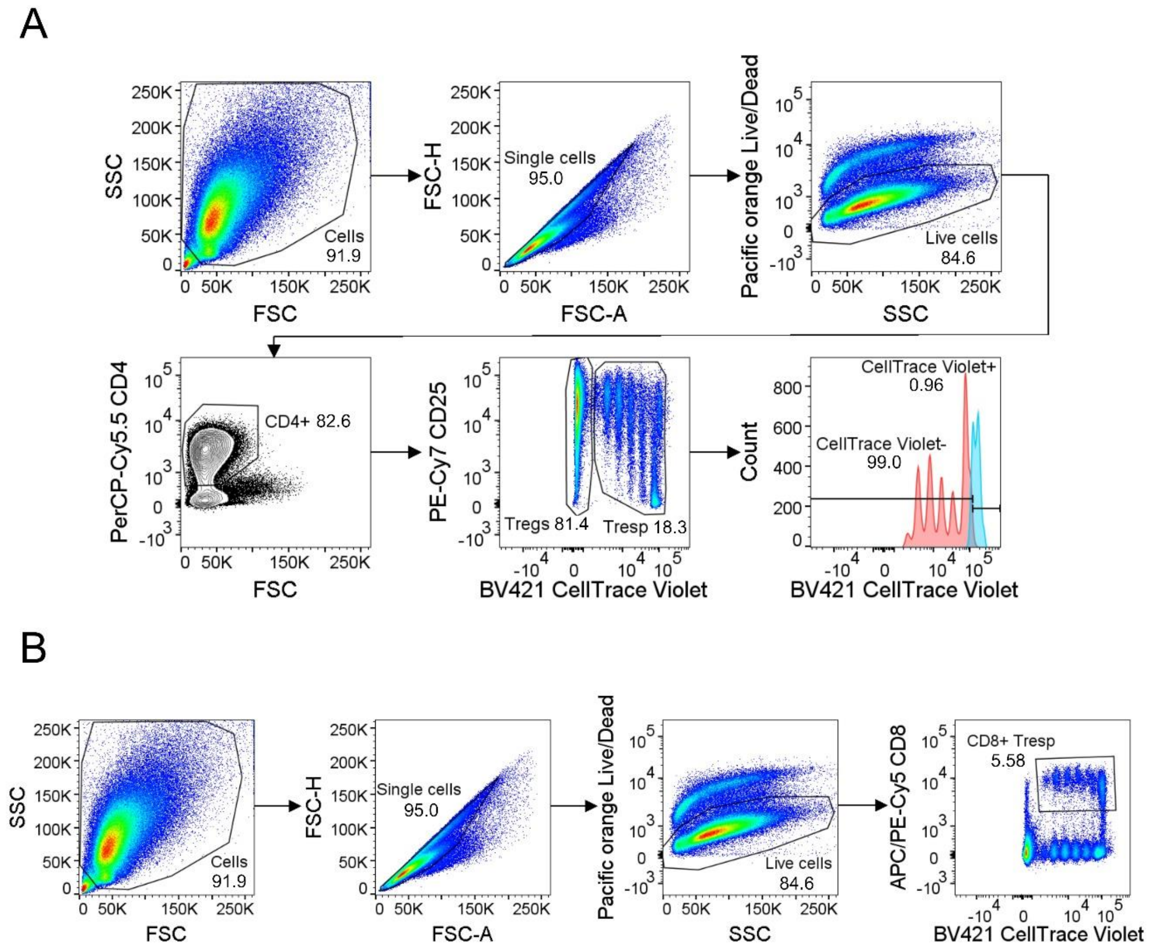

(A) Gating strategy shown for a representative APS-1 patient for CD4+ responder cells. Samples were gated as single, live cells and further as CD4 positive cells. Tresp cells were separated from Tregs by the expression of CD25 and CellTrace Violet. Here, only Tresp cells are stained with the proliferation dye. Tresp cells stained with CellTrace Violet vs. count at day 0 were used to make sure that cells not dividing were included in the responder cell gate. (B) Gating strategy shown for a representative APS-patient for CD8+ responder cells. Samples were gated as single, live cells and further as CD8 positive cells against CellTrace Violet. CD8+ responder cells are separated from CD4+ responder cells using the APC channel.

Figure S19. ***In vitro*** **Treg suppression of CD8+ Tresp cells.** ***Related to main Figure 6.***

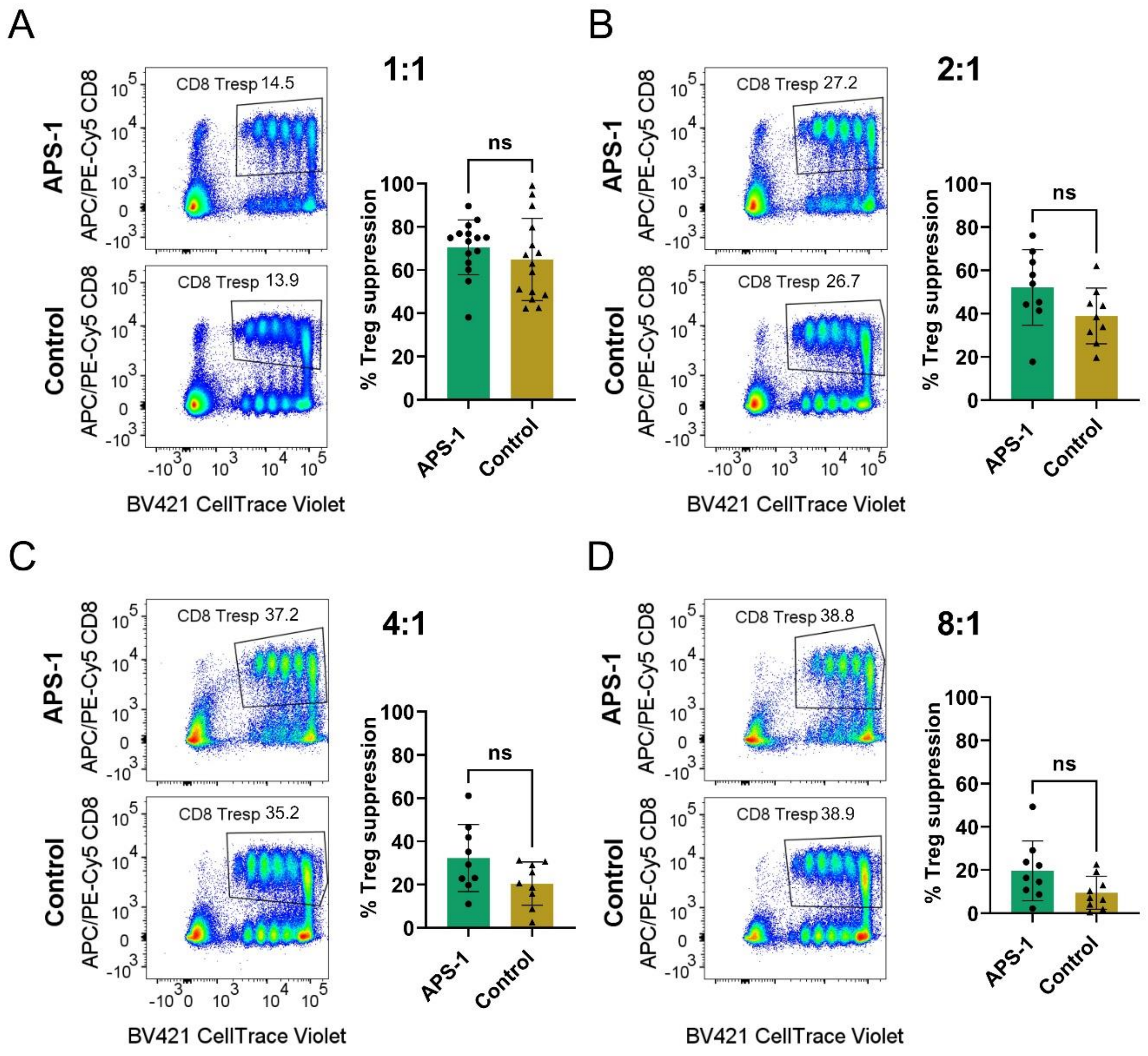

CellTrace Violet labelled CD8+ Tresp cells were co-cultured with Tregs at different ratios for five days in the presence of anti-CD3/CD28 and IL2. (A) Tresp:Treg 1:1, (B) Tresp:Treg 2:1, (C) Tresp:Treg 4:1 and (D) Tresp:Treg 8:1. Flow figures show results from a representative APS-1 patient and a representative healthy control for each ratio. Tresp: Tregs 1:1 includes 15 patients, while 9 patients were tested for all ratios. P-values were determined by an unpaired, parametric t-test. A p-value < 0.05 was considered significant. ns; non-significant.

Figure S20. Proliferation and expansion indices in flow cytometry experiments of responder cell with/without co-culture with Tregs. ***Related to main Figure 6.***

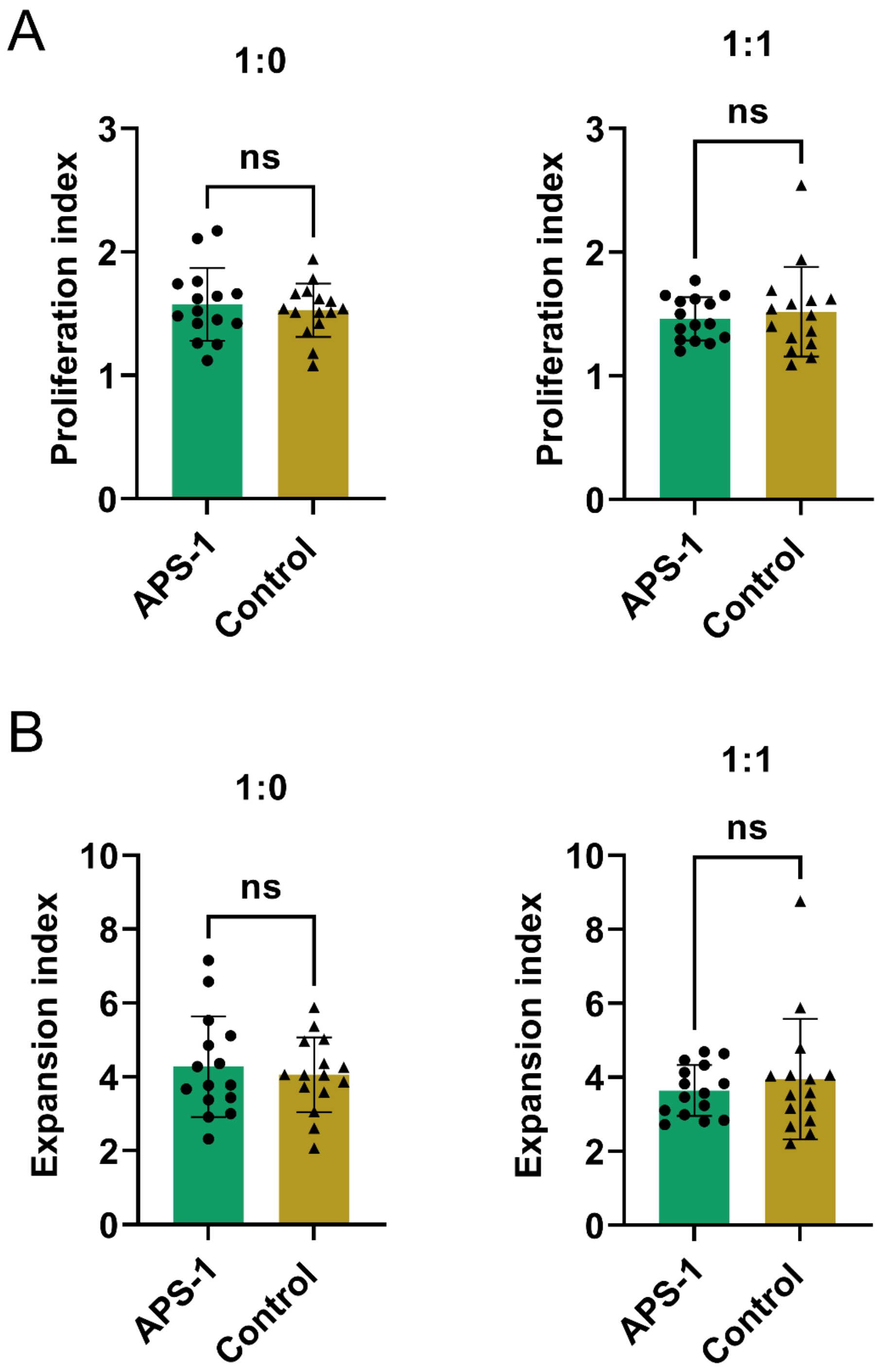

For the Tresp:Treg ratios 1:0 and 1:1, (A) the proliferation and (b) the expansion indexes were examined for patients and controls within the CD4+ responder cell population. P-values were determined by an unpaired, parametric t-test. $p < 0.05$ was considered significant. ns; non-significant.

Figure S21. Measurement of Treg-specific cytokines in expanded Treg suppression assay supernatant. ***Related to main Figure 6.***

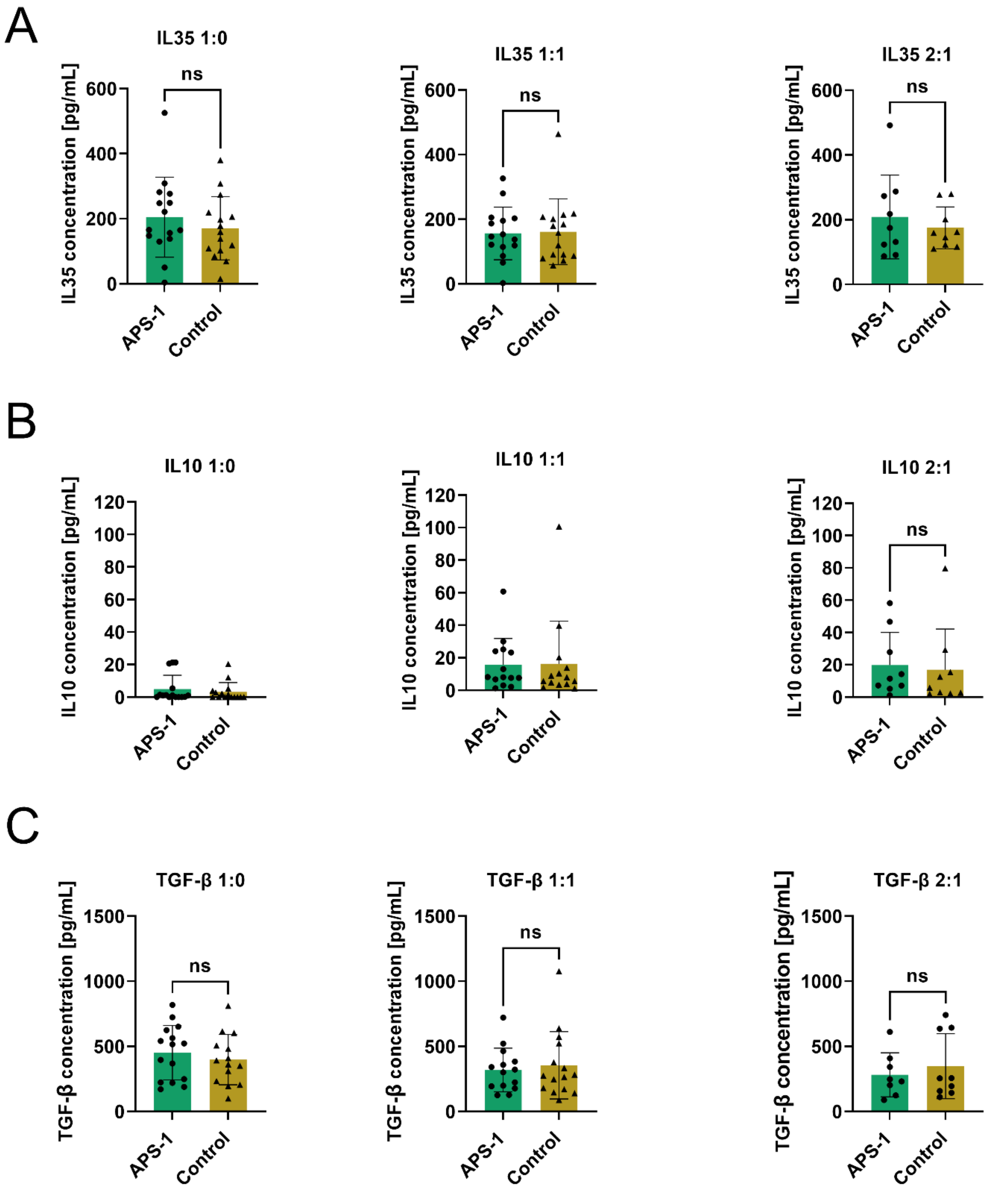

For IL10, 4 patients and 8 controls had non-detectable concentrations for the Tresp:Treg 1:0 samples, while one control sample was outside the range for Tresp:Treg 1:1. These samples were given the minimum detectable dose concentration of 0.03 pg/mL. No statistical analysis was performed on these samples. P-values were determined by an unpaired, parametric t-test. A p-value < 0.05 was considered significant. ns; non-significant.

Table S1A: Flow cytometry panel for Treg characterisation, Treg suppression assay and sorting of freshly isolated Tregs. ***Related to main Figure 4 and 6.***

Marker	Fluorochrome	Clone	Cat. number
CD3 (BD)	V500	UCHT1	561416
CD4 (BioLegend)	PerCP-Cy5.5	RPA-T4	300530
CD25 (BD)	PE-Cy7	2A3	335824
CD45RA (BD)	APC-H7	HI100	560674
CTLA4 (BioLegend)	BV421	BNI3	369606
CD39 (Invitrogen)	PE	eBioA1	12-0399-42
CD31 (BD)	BV785	L133.1	744757
HLA-DR (BD)	BV650	G46-6	564231
CD8 (BD)	PE-Cy5	RPA-T8	555368
FoxP3 (BD)	PE-CF594	236A/E7	563955
Helios (BioLegend)	APC	22F6	137222
CD3 (BioLegend)	FITC	UCHT1	300440
CD25 (BioLegend)	PE	BC96	302606
CD56 (BioLegend)	PE-Cy5	5.1H11	362516
CD14 (BioLegend)	PE-Cy5	M5E2	301864
CD127 (BioLegend)	BV421	A019D5	351310
CD8a (BioLegend)	BV605	RPA-T8	301040
CD21 (BioLegend)	APC	Bu32	354905
CD4 (BioLegend)	AF700	OKT4	317426
CD19 (Miltenyi Biotec)	APC	LT19	130-091-248
CellTrace Violet Cell Proliferation Kit (Invitrogen)	BV421	-	C34557
Live/dead Fixable Yellow Dead Cell Stain kit (Invitrogen)	Q-dot585	-	L34959

Table S1B: Metal-conjugated antibodies used for CyTOF. ***Related to main Figure 5.***

Marker	Metal	Clone	Cat. number
CD103 (Fluidigm)	151Eu	Ber-ACT8	3151011B
CD123 (IL3R) (Fluidigm)	143Nd	6H6	3143014B
CD127 (IL7Ra) (Fluidigm)	149Sm	A019D5	3149011B
CD14 (Invitrogen)	112Cd	MEM15	11514562
CD152 (CTLA4) (Fluidigm)	161Dy	14D3	3161004B
CD161 (Fluidigm)	164Dy	HP-3G10	3164009B
CD19 (Fluidigm)	142Nd	HIB19	3142001B
CD25 (IL2Ra) (Fluidigm)	169Tm	2A3	3169003B
CD27 (Fluidigm)	158Gd	L128	3158010B
CD274 (PD-L1) (Fluidigm)	159Tb	29E.2A3	3159029B
CD278 (ICOS) (Fluidigm)	148Nd	C398.4A	3148021D
CD279 (PD1) (Fluidigm)	155Gd	EH12.2H7	3155009B
CD28 (Fluidigm)	160Gd	CD28.2	3160003B
CD3 (Fluidigm)	170Er	UCHT1	3170001B
CD31/PECAM1 (Fluidigm)	144Nd	WM59	3144023B
CD4 (Fluidigm)	145Nd	RPA-T4	3145001B
CD45 (Fluidigm)	89Y	HI30	3089003B
CD45RA (Fluidigm)	153Eu	HI100	3153001B
CD45RO (Fluidigm)	165Ho	UCHL1	3165011B
CD5 (eBioscience)	166Er	UCHT2	14005982
CD56 (NCAM) (Fluidigm)	163Dy	NCAM16.2	3163007B
CD57 (Fluidigm)	176Yb	HCD57	3176019B
CD66b (Novus Biologicals)	141Pr	G10F5	NBP2-80664
CD69 (Fluidigm)	162Dy	FN50	3162001B
CD8 (Fluidigm)	168Er	SK1	3168002B
HLA-DR (Fluidigm)	174Yb	L243	3174001B
Tigit (Fluidigm)	154Sm	MBSA43	3154016B

Table S2: Primers for qPCR. ***Related to main Figure 2.***

Gene target	Forward primer	Reverse primer
β-actin	5'- GCATGGGTCAGAAGGATTCCT	5'- TCGTCCCAGTTGGTGACGAT
CCR4	5'- CTGTATTCCTTGGTTTTTGT	5'- AGGTCCTTGCCCTCAAGGA
CXCR3	5'- CTACACCGAGGAAATGGG	5'- TGCAACTGCCCAGAAGGGA
FOXP3	5'- ATGCACCAGCTCTCAA	5'- AGTGGGTAGGAGCTCT
GATA3	5'- TCATTAAGCCCAAGCGAAGG	5'- GTCCCCATTGGCATTCCTC
RORγt	5'- TGGACCACCCCCTGCTGAGAAGG	5'-CTTCAATTTGTGTTCTCATGACT
T-bet	5'- GATGCGCCAGGAAGTTTCAT	5'- GCACAATCATCTGGGTCACATT

Table S3: Estimated cell types and cell cycle phase of the "expanded Tregs" from healthy controls (N=8) and APS-1 patients (N=8). ***Related to main Figure 2.***

	Estimated cell types (% of all)				Cell cycle phase (% of all)		
	**Tregs**	**B cells**	**NK cells**	**Th17 like**	**GM2**	**G1**	**S**
**HC1**	73	1	0	26	35	41	24
**HC2**	48	52	0	0	30	52	18
**HC3**	99	1	0	0	52	19	29
**HC4**	99	0	0	0	75	13	12
**HC5**	99	0	1	0	48	23	29
**HC6**	94	1	5	0	47	30	23
**HC7**	98	1	0	1	56	14	30
**HC8**	93	1	6	0	51	16	32
**P5**	92	5	1	1	41	47	13
**P6**	91	9	0	0	43	29	28
**P7**	99	1	0	0	72	9	19
**P8**	100	0	0	0	60	23	17
**P9**	87	0	13	0	41	35	24
**P10**	99	1	0	0	53	17	31
**P11**	98	0	0	2	82	7	12
**P12**	100	0	0	0	39	40	21

Tregs linage based on the RNA expression of TNFRSF4, SPOCK2, CD28, CD3D and G, CD4 and FOXP3. Natural killer (NK) cells based on positivity for FCER1GA/3A, KIR2DL3/2DL1, STSW, GNLY, CCL3/4, TRDC and PRF1 expression. B cells based on positive expression of Jchain, CCL17/22, IGHM/KC/HG1/HA1, CD79A, MS4A1 and CXCL10. Outliers with high percentage of other cell types than Tregs are marked in red.

Table S4: Frequency of cell subsets in the expanded Tregs estimated by mass cytometry for APS-1 patients (N=17) and healthy controls (N=17). ***Related to main Figure 5.***

Mass cytometry on expanded Tregs

	**Immune cell subsets (% of CD45 if not otherwise is stated)**				
	**Tregs CD25+CD127-/CD4+**	**Monocytes CD14+**	**B cells CD19+**	**NK cells CD56+**	**Cytotoxic T cells CD8+**
**C1**	89	0	1	0	0
**C2**	80	0	1	7	0
**C3**	90	0	2	0	0
**C4**	93	1	2	1	0
**C5**	79	0	0	1	0
**C6**	77	0	0	0	0
**C7**	78	0	0	0	0
**C8**	73	0	2	2	1
**P5**	92	0	1	1	0
**P6**	86	0	2	0	0
**P7**	79	2	2	1	0
**P8**	84	1	2	0	0
**P9**	70	0	0	0	0
**P10**	74	0	0	1	0
**P11**	74	0	0	1	0
**P12**	69	0	0	2	0
**C9**	83	0	0	0	2
**C10**	85	0	3	1	1
**C11**	77	0	0	0	0
**C12**	82	0	0	0	0
**C13**	82	0	0	0	0
**C14**	85	0	0	0	0
**C15**	85	0	5	1	1
**C16**	93	0	0	0	18
**C17**	83	0	6	1	2
**P13**	78	0	0	0	0
**P14**	76	0	0	0	0
**P15**	80	0	0	0	0
**P16**	82	0	0	0	0
**P17**	76	0	0	0	0
**P18**	76	0	0	1	1
**P19**	76	0	0	0	0
**P20**	80	0	0	0	0
**P21**	85	0	0	0	0

Outliers with high frequencies (>3%) of other cell populations than Tregs (CD4+ CD25+ CD127-) are marked in red.

Table S9A: Overview of patients and experiments. ***Related to main Table 1 and all Figs.***

Patient	Sex[a]	Single-cell sequencing	Suppression assay	CyTOF characterization	Flow characterization
1	F	x			
2	F	x			
3	F	x			
4	F	x			
5	M	x	x	x	x
6	M	x	x	x	x
7	F	x	x	x	x
8	F	x	x	x	x
9	M	x	x	x	x
10	F	x	x	x	x
11	M	x	x		
12	M	x	x	x	x
13	F	x	x	x	x
14	M		x	x	x
15	M		x	x	x
16	M		x	x	x
17	F		x	x	x
18	F		x	x	x
19	F		x	x	x
20	M			x	x
21	F			x	x
22	F			x	x

Table S9B: Overview of healthy controls and experiments.

Control	Sex[a]	Age (years)	Single-cell sequencing	Suppression assay	CyTOF characterization	Flow characterization
1	F	39	x			
2	F	61	x			
3	F	63	x			
4	F	30	x			
5	M	58	x	x	x	x
6	M	54	x	x	x	x
7	F	47	x	x	x	x
8	F	47	x	x	x	x
9	M	35	x	x	x	x
10	F	22	x	x		
11	M	54	x	x		
12	M	30	x	x	x	x
13	F	62	x	x		
14	F	29			x	x
15	F	60			x	x
16	M	29		x	x	x
17	M	34		x	x	x
18	M	36			x	x
19	M	34		x	x	x
20	F	70		x	x	x
21	F	42			x	
22	F	59		x	x	
23	F	20			x	x
24	F	60		x	x	x

[a] F, female; M, male.
